# Multiplex genome engineering in *Clostridium beijerinckii* NCIMB 8052 using CRISPR-Cas12a

**DOI:** 10.1038/s41598-023-37220-y

**Published:** 2023-06-22

**Authors:** Constantinos Patinios, Stijn T. de Vries, Mamou Diallo, Lucrezia Lanza, Pepijn L. J. V. Q. Verbrugge, Ana M. López-Contreras, John van der Oost, Ruud A. Weusthuis, Servé W. M. Kengen

**Affiliations:** 1 Laboratory of Microbiology, Wageningen University and Research, Stippeneng 4, 6708 WE Wageningen, The Netherlands; 2 Bioprocess Engineering, Wageningen University and Research, Droevendaalsesteeg 1, 6708 PB Wageningen, The Netherlands; 3 Bioconversion Group, Wageningen Food and Biobased Research, Wageningen University and Research, Bornse Weilanden 9, 6708 WG Wageningen, The Netherlands

**Keywords:** Biotechnology, Microbiology, Molecular biology

## Abstract

*Clostridium* species are re-emerging as biotechnological workhorses for industrial acetone–butanol–ethanol production. This re-emergence is largely due to advances in fermentation technologies but also due to advances in genome engineering and re-programming of the native metabolism. Several genome engineering techniques have been developed including the development of numerous CRISPR-Cas tools. Here, we expanded the CRISPR-Cas toolbox and developed a CRISPR-Cas12a genome engineering tool in *Clostridium beijerinckii* NCIMB 8052. By controlling the expression of FnCas12a with the xylose-inducible promoter, we achieved efficient (25–100%) single-gene knockout of five *C. beijerinckii* NCIMB 8052 genes (*spo0A*, *upp*, *Cbei_1291*, *Cbei_3238*, *Cbei_3832*). Moreover, we achieved multiplex genome engineering by simultaneously knocking out the *spo0A* and *upp* genes in a single step with an efficiency of 18%. Finally, we showed that the spacer sequence and position in the CRISPR array can affect the editing efficiency outcome.

## Introduction

*Clostridium beijerinckii* NCIMB 8052, a gram-positive, spore-forming, anaerobic bacterium, is a member of the acetone–butanol–ethanol (ABE) producing *Clostridium* species. ABE fermentation has a considerable industrial history as it played a major role during the twentieth century and especially during World War I for the production of acetone (to produce cordite/gunpowder), butanol, lacquer solvents and jet fuel, before being outcompeted by petrochemical processes^1–4^. Due to economic and environmental reasons and due to advances in biotechnology, sustainable ABE fermentation using *Clostridium* species has re-emerged^5^.

**Figure 1:**
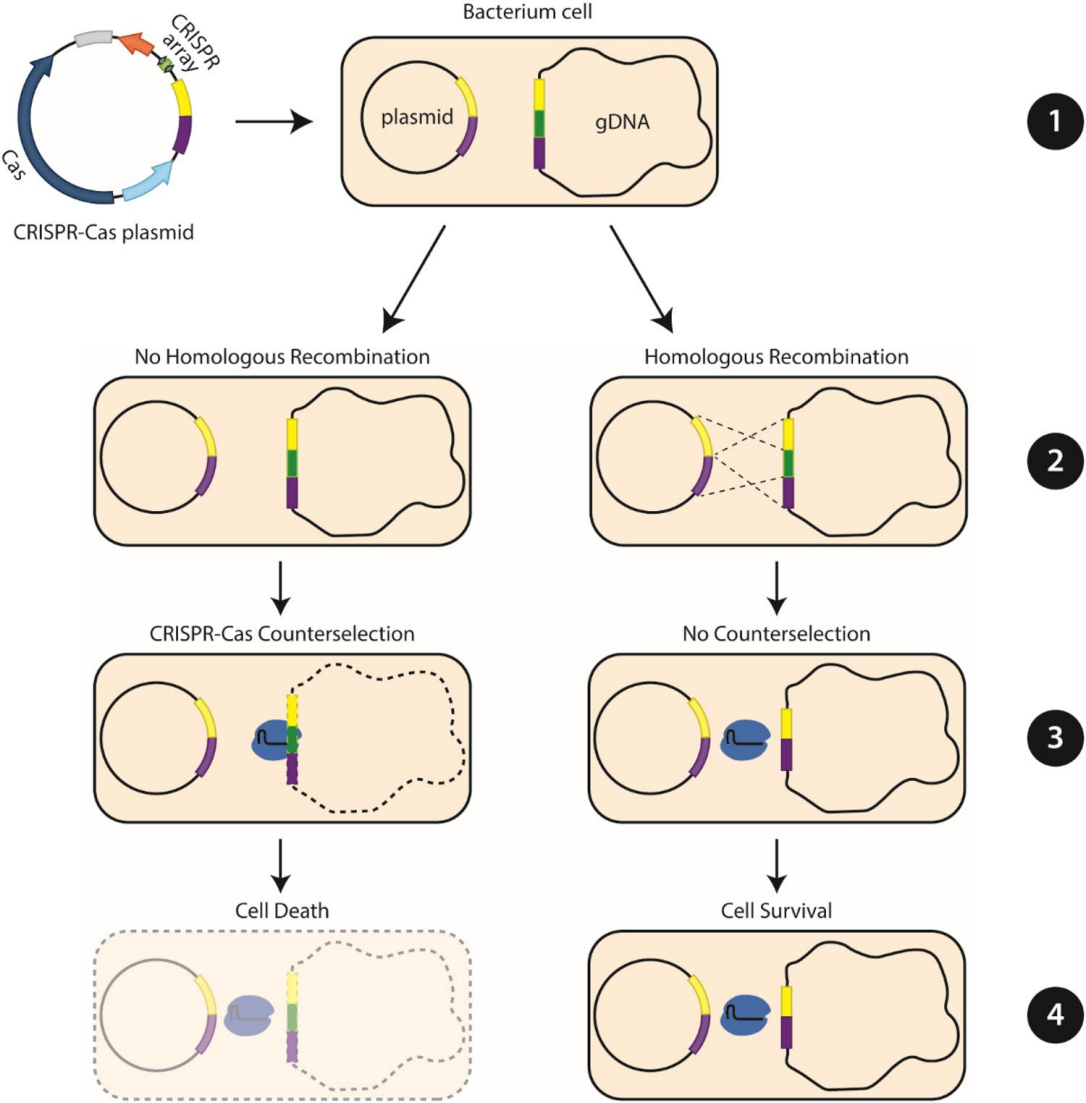
Principle of CRISPR-Cas-mediated counterselection for efficient selection of edited bacterial cells. (1) A plasmid DNA bearing the CRISPR-Cas system (CRISPR array and Cas protein) and homologous arms (depicted with colours yellow and purple) to knock-out the gene of interest (depicted with green and flanked by the homologous arms on the genomic DNA; gDNA), is transformed to the bacterium of interest. (2) The bacterium genome will either recombine with the homology arms of the transformed plasmid to remove and edit the target site (right path; Homologous Recombination) or, it will remain unedited and retain its wild-type genotype (left path; No Homologous Recombination). (3) Expression of CRISPR-Cas (indicated with blue) will target and cut the unedited wild-type genome (left path) due to the presence of a targeting site in the gene of interest, whereas it will not target the edited genome (right path) due to the removal of the target site through homologous recombination. (4) Bacterial cells that carry an unedited, wild-type genome will die from the dsDNA break caused by CRISPR-Cas whereas bacterial cells carrying an edited genome will not be targeted by CRISPR-Cas and will survive.

To take advantage of the industrial potential of *Clostridium* species, several genome engineering tools have been developed^6^. The genome engineering tools can be broadly divided into the ones which rely on homologous recombination (HR) based allelic exchange and the ones which depend on group II intron-retargeting mutagenesis (TargeTron/ClosTron)^7,8^. Whilst the group II intron-retargeting mutagenesis generally allows quick and efficient genome engineering, it has several disadvantages including the inability to target genes smaller than 400 bp, it interrupts rather than deletes the target of interest, it frequently relies on the genomic integration of antibiotic resistance genes and the intron may be spliced back out by its associated intron-encoding protein^9^. On the other hand, HR-based techniques enable the generation of scarless and complete deletion mutants and they do not rely on the integration of antibiotic resistance genes in the genome of the target organism. For high accuracy, however, HR should be combined with an efficient counterselection mechanism such as CRISPR-Cas, that allows for efficient elimination of unedited cells through the generation of double stranded DNA (dsDNA) breaks in the genome of the bacterium^10^. Due to the absence of efficient dsDNA break repairing mechanisms (e.g. non-homologous end joining; NHEJ), only the recombined cells can survive the detrimental effect of dsDNA breaks generated by CRISPR-Cas (Fig. 1). Following this requirement, CRISPR-Cas in combination with HR has been used to achieve high editing efficiencies in various *Clostridia* species as summarized by McAllister and Sorg (2019)^6^.

To date, several CRISPR-Cas-based tools have been developed for single gene editing in *C. beijerinckii*, whereas multiplex gene editing in this species has never been demonstrated before^9,11–16^. Most of the developed CRISPR-Cas tools rely on the Cas9 nuclease and only a small fraction is based on the Cas12a nuclease. The preference towards Cas9 is probably due to a first-comer effect and due to its successful application in various organisms^17^. However, Cas12a has distinctive advantageous features over Cas9 including smaller size (Cas12a: ~ 1300 amino acids, Cas9: ~ 1600 amino acids) and recognition of a T-rich 5′-(T)TTV-3′ PAM site at the 5′ end of the protospacer sequence which increases the number of target sites in AT-rich organisms like *Clostridia* (~ 30% GC-content)^18^. In addition, Cas12a can process its own crRNA array due to its RNase activity; a feature that makes Cas12a ideal for multiplex genome engineering as the transcription of a single CRISPR array expressed by a single promoter is the only requirement for the generation of multiple crRNAs^18^.

In this study, we used the FnCas12a nuclease to create single- and multi-gene deletions in *C. beijerinckii* NCIMB 8052. Depending on the target gene, single-gene knockout efficiencies varied from 25 to 100%. Multiplex (two gene) deletion was also achieved in one step with a knockout efficiency of 18%. Spacer sequence and position in the CRISPR array affected the multiplex knockout efficiency, revealing potential limitations and room for improvement.

## Results and discussion

### Markerless deletion of *spo0A* through inducible expression of FnCas12a

A previous report showed successful single-gene genome engineering of *C. beijerinckii* NCIMB 8052 using AsCas12a^15^. The AsCas12a used in that study was derived from pDEST-hisMBP-AsCpf1-EC (Addgene plasmid #79,007) which has a codon optimized nucleotide sequence for *E. coli*. Since *C. beijerinckii* NCIMB 8052 and *E. coli* differ in genomic GC- content (30% vs. 51%, respectively), the use of the *E. coli* optimized AsCpf1 in *C. beijerinckii* NCIMB 8052 may have retarded translation speed and fidelity (Fig. S1)^19,20^. Considering codon usage as an important factor for successful protein expression and folding, we reasoned that we should use a *Cas12a* gene that matches the codon usage of *C. beijerinckii* NCIMB 8052 and also follows the same translation speed and fidelity^19^. To this end, we have chosen the wild type FnCas12a nuclease based on its low GC percentage (30%) and matching codon usage for *C. beijerinckii* NCIMB 8052 (Fig. S1).

**Figure 2:**
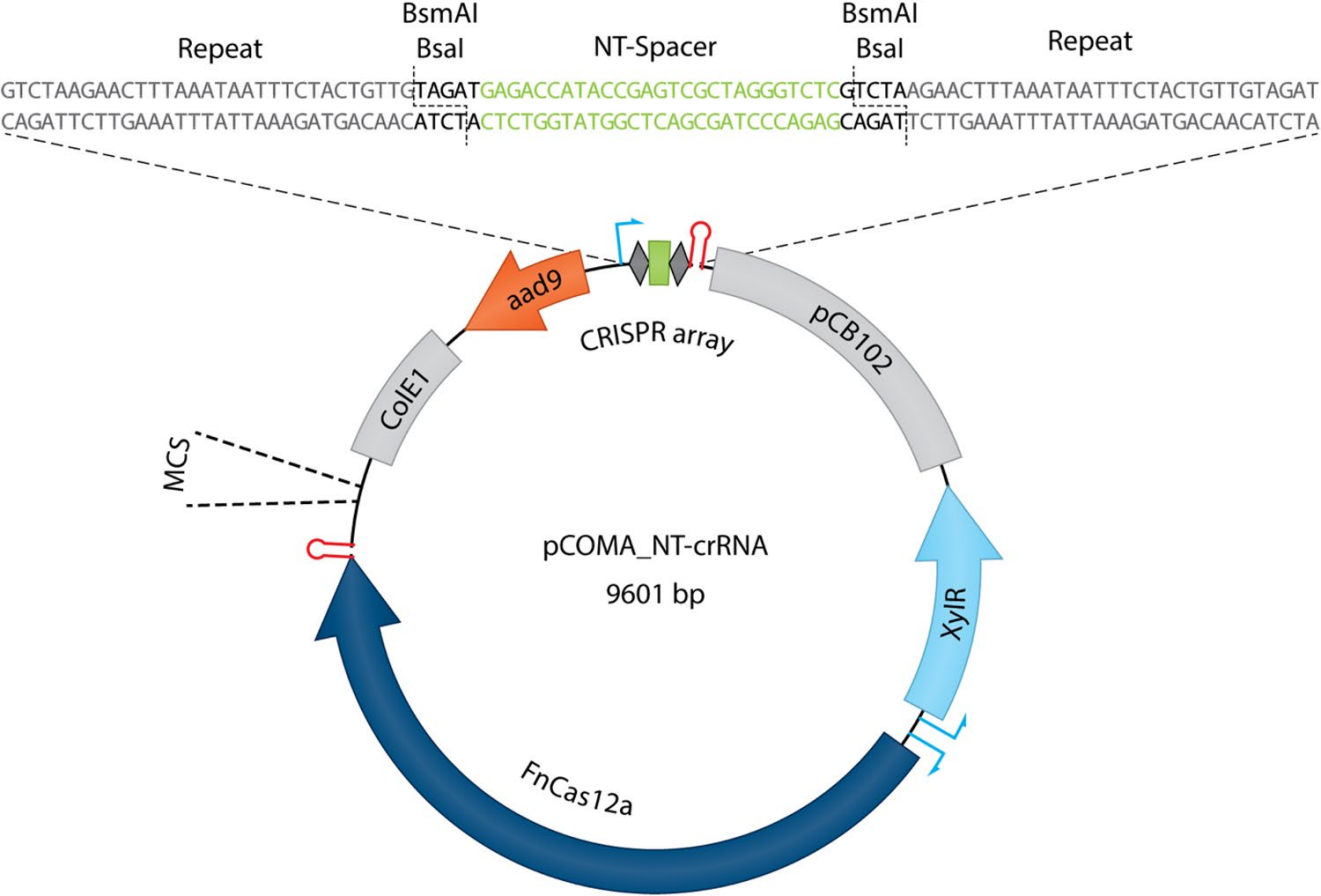
Backbone of the pCOMA plasmid series. At the top of the plasmid the CRISPR array is shown, where a non-targeting (NT) spacer can be conveniently replaced using Golden Gate. Homologous arms can be inserted at the multiple cloning site (MCS) using Gibson Assembly. ColE1: Origin of replication for *E. coli*; aad9: Spectinomycin resistance gene; CRISPR array: composed by the Thl promoter, a non-targeting (NT) spacer (green rectangle) flanked by two repeats (dark grey rhombuses) and the Thl terminator; pCB102: Origin of replication for *C. beijerinckii* NCIMB 8052; XylR: Xylose repressor expressed by the XylR promoter; FnCas12a: *Francisella novicida* Cas12a flanked by the XylB inducible promoter and the Fdx terminator.

To develop a simple genome engineering tool for *C. beijerinckii* NCIMB 8052, a single plasmid approach was used containing the *FnCas12a* gene, the CRISPR array (repeat-spacer-repeat) with an insertion site for easy exchange of the spacer through Golden Gate and a multiple cloning site (MCS) to insert the homology arms and facilitate gene knockout (Fig. 2). To control the expression of FnCas12a and avoid potential cell toxicity due to the constitutive expression of the CRISPR-Cas system^12,21–24^, we chose to use the xylose-inducible promoter derived from *C. difficile*^16,25,26^. The use of the xylose-inducible system has a dual function in our setup as it can serve both as the inducer molecule for the expression of FnCas12a but also as carbon- and energy-source for growth. Therefore the use of glucose in the growth medium can be omitted and the effect of potential catabolite repression can be avoided^26^. To express the crRNA, we chose to use the endogenous strong constitutive thiolase promoter (ThlP) from *C. beijerinckii* NCIMB 8052.

As a proof-of-principle, we selected the well-characterized *spo0A* (Cbei_1712) gene as a knockout target. The Δ*spo0A* strain has a distinctive morphological and metabolite production phenotype, making it easy to identify *spo0A* knockouts^27–29^. To this end, plasmids pCOMA_NT-crRNA (non-targeting control), pCOMA_spo0A-crRNA (targeting), pCOMA_NT-crRNA_Spo0AHA (non-targeting control with homology arms) and pCOMA_spo0A-crRNA_Spo0AHA (targeting with homology arms) were constructed and used for the transformation of *C. beijerinckii* NCIMB 8052 cells. Transformed cells were screened for the presence of the plasmid through colony PCR and were then grown for 48 h in mCGM-G liquid medium, after which 100 μL of the culture were plated on mCGM-G solid medium (as a control) and 100 μL of the culture on mCGM-X solid medium to induce the expression of FnCas12a and, hence, counterselect the wild type (WT) from the edited cells (Fig. 1).

**Figure 3:**
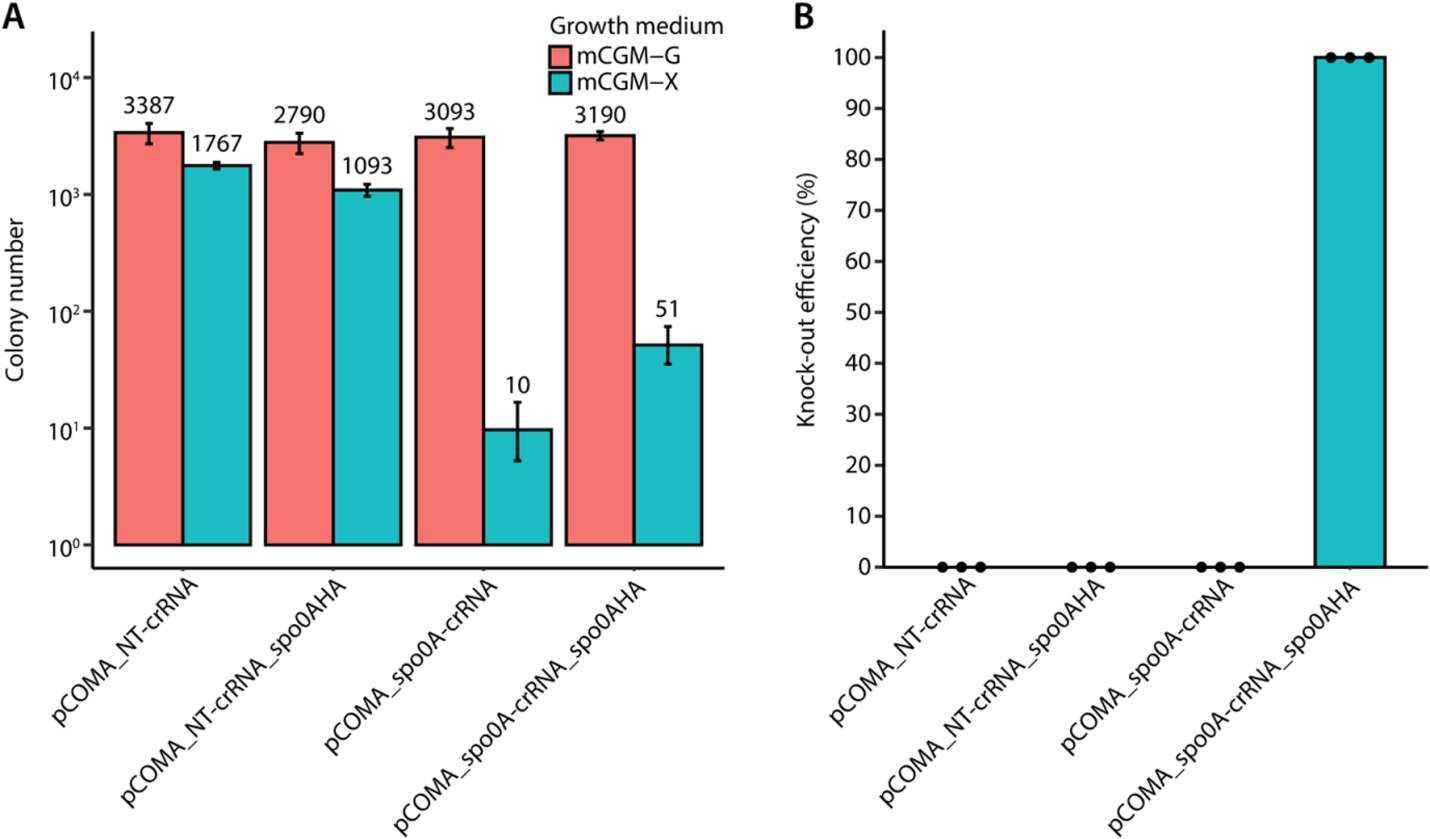
Inducible counterselection and *spo0A* knockout in *C. beijerinckii* NCIMB 8052. *C. beijerinckii* NCIMB 8052 cells were transformed either with the pCOMA_NT-crRNA and pCOMA_NT-crRNA_Spo0AHA non-targeting plasmids (negative controls), the pCOMA_spo0A-crRNA targeting plasmid (positive control) or the pCOMA_spo0A-crRNA_Spo0AHA plasmid. Transformants were plated on mCGM-G (no-induction) or on mCGM-X (induction of FnCas12a). This experiment was performed in biological triplicates. The error bars in (**A**) show the standard deviation. (**A**) Colony number obtained after plating the transformants on the appropriate medium. The numbers above each bar indicate the average obtained amount of colonies. (**B**) *spo0A* knockout efficiency determined by screening eight colonies from each replicate. Dots represent the knockout efficiency from each replicate.

Transformants plated on mCGM-G solid medium showed comparable numbers of colonies (approximately 10^3^), regardless of the transformed plasmid (Fig. 3A). Similarly, transformants carrying a non-targeting spacer, led to approximately 10^3^ colonies when plated on mCGM-X. In contrast, transformants carrying a targeting spacer and plated on mCGM-X showed fewer colonies compared to the non-targeting controls. As expected, transformation with pCOMA_spo0A-crRNA resulted in very few colonies (~ 10), which shows the functionality of FnCas12a to successfully target and cleave the genome of *C. beijerinckii* NCIMB 8052, leading to cell death. The presence of a small number of colonies may be attributed to PAM, spacer, protospacer, crRNA or FnCas12a mutants that could escape the counterselective properties of FnCas12a. A reduced number of colonies (~ 51) compared to the non-targeting controls, but 5 × higher than pCOMA_spo0A-crRNA was observed when the pCOMA_spo0A-crRNA_Spo0AHA plasmid was used.

To assess whether the colonies obtained on mCGM-X were successful *spo0A* knockouts, we screened eight colonies (if present) from each biological replicate (24 in total) through colony PCR. As expected, the non-targeting controls showed a 0% knockout efficiency represented by a WT genotype of 1866 bp amplicons (Figs. 3B and S2). Similarly, the obtained pCOMA_spo0A-crRNA colonies showed a WT genotype (0% knockout efficiency), further supporting that the obtained colonies are escapees. Intriguingly, obtained colonies containing the pCOMA_spo0A-crRNA_Spo0AHA had a 100% knockout efficiency with a Δ*spo0A* genotype corresponding to 1044 bp amplicons (Figs. 3B and S2). Three mutant colonies were further assessed for the deletion of *spo0A* by Sanger sequencing, confirming the scarless deletion of *spo0A* (Fig. S3, Table 1 and Table S6). Our obtained 100% (24 out of 24 colonies tested) knockout efficiency is equal to the previously reported knockout efficiencies of *spo0A* using CRISPR-Cas9 (100%; 5 out of 5 colonies tested)^9^ and CRISPR-AsCas12a (100%; 24 out of 24 colonies tested)^15^, demonstrating the functionality of our tool.

**Table 1:** Strains used or generated in this study.

Strain	Genotype	Source	Benchling link
*Escherichia coli* NEB® 5-alpha	*fhuA2 Δ(argF-lacZ)U169 phoA glnV44 Φ80 Δ(lacZ)M15 gyrA96 recA1 relA1 endA1 thi-1 hsdR17*	NEB	N/A
*Clostridium beijerinckii* NCIMB 8052	Wild type	^36^	N/A
*C. beijerinckii* NCIMB 8052 Δ*spo0A*	Δ*spo0A* (*Cbei_1712*)	This study	https://benchling.com/s/seq-urQiulVV7bnnWSpFoAwz?m=slm-eUXfGmPF5u2sF5NuTqiY
*C. beijerinckii* NCIMB 8052 Δ*upp*	Δ*upp* (*Cbei_0408*)	This study	https://benchling.com/s/seq-1JOwdJt8QWOzYhCDVSTl?m=slm-4JyiiWkuvvRC1rhpac4c
*C. beijerinckii* NCIMB 8052 Δ*spo0A*, Δ*upp*	Δ*spo0A* (*Cbei_1712*), Δ*upp* (*Cbei_0408*)	This study	N/A
*C. beijerinckii* NCIMB 8052, Δ*Cbei_1291*	Δ*Cbei_1291*	This study	https://benchling.com/s/seq-51mieTUESpOi1YjGlySN?m=slm-LklOYAluMlSUJB9Vs7P8
*C. beijerinckii* NCIMB 8052, Δ*Cbei_3238*	Δ*Cbei_3238*	This study	https://benchling.com/s/seq-J8n9JPZdYYAFZWkztce4?m=slm-Qe2GaA2L75nmhpdaXVGn
*C. beijerinckii* NCIMB 8052, Δ*Cbei_3932*	Δ*Cbei_3932*	This study	https://benchling.com/s/seq-N60RTFAV4KA28JkzX1Aj?m=slm-IDRoJQUoZQknBmZeZBhF

### Δ*spo0A C. beijerinckii* NCIMB 8052 shows retarded growth, elimination of solvent production and increased production of acids

**Figure 4:**
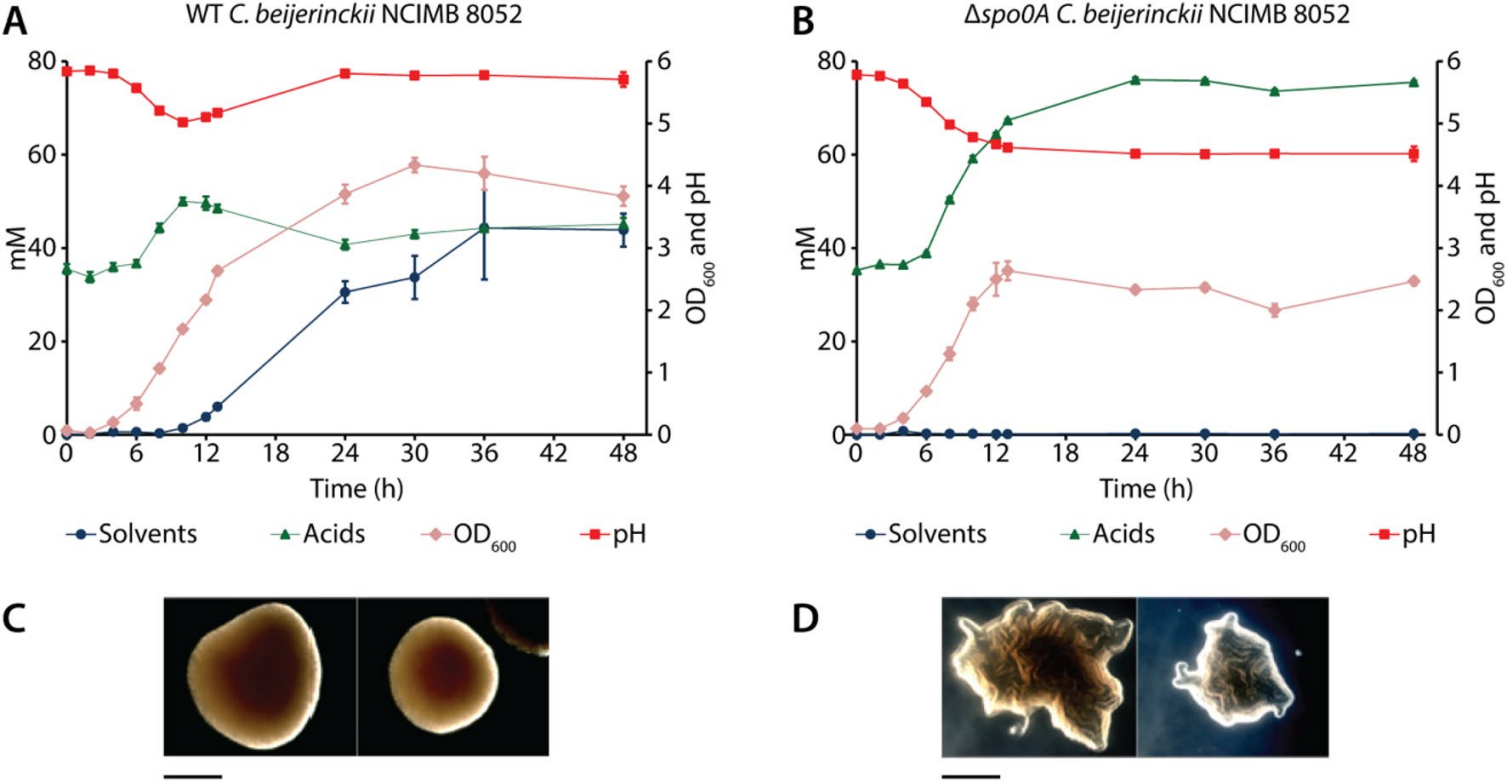
Fermentation profile and morphology of WT and Δ*spo0A C. beijerinckii* NCIMB 8052 strains. (**A**) Fermentation profile of WT *C. beijerinckii* NCIMB 8052. (**B**) Fermentation profile of Δ*spo0A C. beijerinckii* NCIMB 8052. The error bars in (**A**) and (**B**) indicate the standard deviation calculated from a triplicate experiment. Solvents represent acetone, butanol and ethanol. Acids represent acetate, butyrate and lactate. (**C**) Colony morphology of WT *C. beijerinckii* NCIMB 8052. (**D**) Colony morphology of Δ*spo0A C. beijerinckii* NCIMB 8052. The scale bar below (**C**) and (**D**) is equal to 1 mm in length.

As previously described^27–29^, Δ*spo0A C. beijerinckii* strains show a distinctive phenotype which includes the elimination of solvent production, increased acid production and altered colony morphology. To assess whether our Δ*spo0A C. beijerinckii* NCIMB 8052 mutants show the described phenotypic characteristics, we selected three Δ*spo0A* colonies and subjected them to plasmid curing. Three cured Δ*spo0A C. beijerinckii* NCIMB 8052 colonies and three WT *C. beijerinckii* NCIMB 8052 colonies were then grown in GAPES medium for 48 h and the fermentation products were analysed (Fig. 4A,B). Raw data of the fermentation and growth profiles are also presented in Supplementary Tables 1, 2, 3 and 4.

During the first 8 h of incubation, the Δ*spo0A* and WT strains performed similarly, as the acids were produced to equimolar amounts, accompanied with the characteristic pH drop from pH 6.0 to around pH 5.0. However, after 8 h of incubation, the growth and product formation of the Δ*spo0A* and WT strains differed considerably. The WT strain ceased the production of acids and the production of solvents was initiated. In contrast, the Δ*spo0A* strain did not assimilate the produced acids after 8 h of growth, resulting in an increased production of acids and the absence of solvent production. Lastly, the distinct, snowflake-like colony phenotype was apparent for the Δ*spo0A* colonies whereas the WT colonies showed the typical round and smooth shape (Fig. 4C,D).

### Establishing single deletions of various *C. beijerinckii* NCIMB 8052 genes

To assess the applicability and knockout efficiency of our tool to other genes (other than the *spo0A*), we sought to delete four genes at different genomic loci: *Cbei_0408* (*upp*; 630 bp), *Cbei_1291* (987 bp), *Cbei_3238* (888 bp) and *Cbei_3932* (813 bp). An identical protocol as the one described for *spo0A* deletion was followed and obtained colonies were screened for mutants through colony PCR and Sanger sequencing (Table 1 and Table S6).

**Figure 5:**
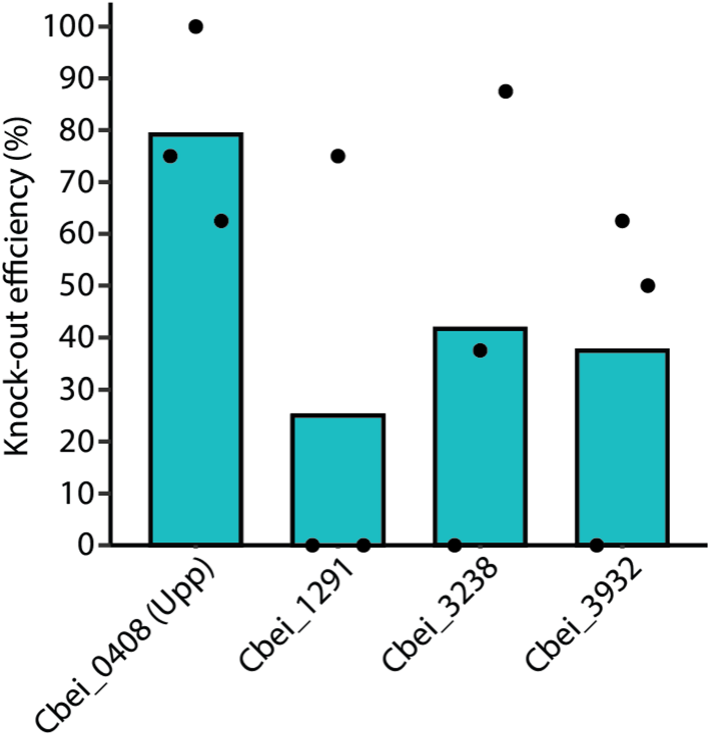
Single-gene knockout of multiple genes in *C. beijerinckii* NCIMB 8052. *Cbei_0408* (*upp*), *Cbei_1291*, *Cbei_3238* and *Cbei_3932* were targeted for knockout. The average knockout efficiency for each gene is: *Cbei_0408* (79.17%), *Cbei_1291* (25%), *Cbei_3238* (41.67%) and *Cbei_3932* (37.5%). This experiment was performed in biological triplicates. The knockout efficiency was determined by screening eight colonies from each replicate through colony PCR using the primers listed in Table S5. Dots represent the knockout efficiency from each replicate.

In contrast to the *spo0A* knockouts, the knockout efficiency varied amongst the selected genes. The highest knockout efficiency was observed for *upp* (79%), followed by *Cbei_3238* (42%), *Cbei_3932* (38%) and *Cbei_1291* (25%) (Figs. 5 and S4). More intriguingly, we clearly observed different knockout efficiencies between the biological replicates of transformants carrying the same plasmid variant. For example, for *Cbei_1291*, two out of the three biological replicates showed 0% knockout efficiency, whereas one of the replicates showed 75% knockout efficiency (Fig. S4). The inconsistency in editing amongst the replicates was also observed for *Cbei_3238* and *Cbei_3932* where at least one of the replicates showed 0% knockout efficiency. Nonetheless, one of the replicates for *Cbei_3238* showed 88% knockout efficiency and one of the replicates for *Cbei_3932* showed 63% knockout efficiency. Knocking out the *upp* gene was more consistent as biological replicates varied between 63 and 100% knockout efficiency.

The variable knockout efficiency amongst the different genes may be attributed to the selection of a good or bad spacer^30^ which is often attributed to the secondary structure of the crRNA, the GC content of the protospacer and the spacer, and the melting temperature of the spacer–protospacer pairing^31,32^. However, the variable knockout efficiency amongst the biological replicates targeting the same gene could be due to early escapees which dominated the culture during the 48 h growth before inducing the expression of FnCas12a for counterselection. Unfortunately, we did not analyse the cause of escapees but potential reasons may include mutations at the spacer or protospacer, at the PAM sequence or the *FnCas12* gene sequence.

### Multiplex gene knockout in *C. beijerinckii* NCIMB 8052

Single-gene knockout in *C. beijerinckii* NCIMB 8052 was previously achieved using CRISPR-AsCas12a^15^. However, multiplex genome editing was never demonstrated before. To show that multiplex gene knockout is possible in *C. beijerinckii* NCIMB 8052, we sought to knockout the *spo0A* and *upp* genes in a single step (i.e., in one transformation event) using our established xylose-inducible system as described above.

To target two genomic sequences (*spo0A* and *upp*) with FnCas12a, two spacers (one for each target) were introduced into the CRISPR array (Table S5). Since the spacer position in the CRISPR array can affect the editing efficiency^33^, we constructed a CRISPR array where the spo0A spacer preceded the upp1 spacer (spo0A-upp1) and a CRISPR array where the upp1 spacer preceded the spo0A spacer (upp1-spo0A). In addition, to further assess the effect of changing one of the targeting spacers with another spacer that targets the same gene but in a different position within the same gene, we replaced the upp1 spacer with the upp2 spacer. The knockout efficiency using upp2 alone was near to 100%, making it a suitable spacer for this experiment (Fig. S5). Similarly, a CRISPR array where the spo0A spacer preceded the upp2 spacer (spo0A-upp2) and a CRISPR array where the upp2 spacer preceded the spo0A (upp2-spo0A) were constructed (Table S5).

**Figure 6:**
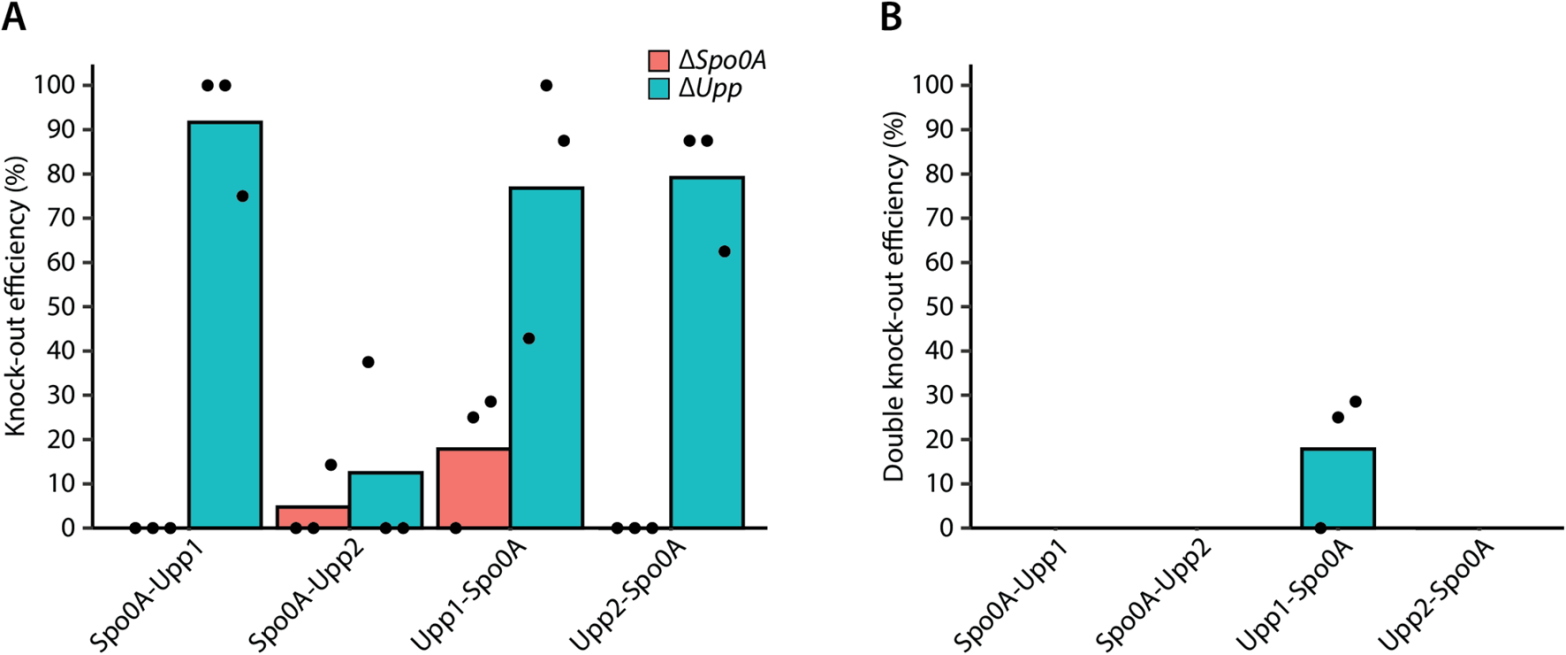
Multiplex gene knockout. The *spo0A* and *upp* genes were targeted simultaneously for knockout in a single step. Different CRISPR arrays were used with either the spo0A spacer preceding the upp spacer or the other way around. Two different upp spacers were used, designated as upp1 and upp2. (**A**) Knockout efficiency of either the *spo0A* or the *upp* gene using the different CRISPR arrays. (**B**) Double knockout efficiency of the *spo0A* and *upp* genes using the different CRISPR arrays. The knockout efficiency was determined by screening eight colonies from each replicate through colony PCR using the primers listed in Table S5. Dots represent the knockout efficiency from each replicate.

As expected, knockout efficiencies varied between the different CRISPR array variants (Fig. 6). The highest knockout efficiency for *upp* (91%) was observed when the spo0A-upp1 CRISPR array was used, whereas the lowest knockout efficiency (13%) was observed when the spo0A-upp2 CRISPR array was used. While the spo0A-upp1 CRISPR array showed the highest knockout efficiency for *upp*, 0% knockout efficiency was observed for *spo0A*. In contrast, by switching the position of the spo0A and upp1 spacers (i.e., from spo0A-upp1 to upp1-spo0A), 18% knockout efficiency was observed for *spo0A*. The knockout efficiency of *upp* dropped to 77% when the upp1-spo0A CRISPR array was used, indicating that the position of the upp1 spacer in the array does not affect largely the knockout efficiency of *upp*. Following, the knockout efficiency of *upp* was reduced from 92 to 13% when the upp1 spacer was substituted with the upp2 spacer and when the spo0A spacer was the first spacer of the CRISPR array. However, the substitution of upp1 to upp2 yielded some (5/24) successful spo0A knockouts, although most (4/5) of them were mixed colonies, as indicated in Fig. S6. Important to note is that half (12/24) of the screened colonies had a mixed (WT and Δ*Upp*) genotype.

In summary, we achieved multiplex gene knockout in *C. beijerinckii* NCIMB 8052, although with low editing efficiency. Clean (i.e., without the presence of mixed colonies) double *spo0A* and *upp* mutants were observed only when the upp1-spo0A CRISPR array was used. This observation is not surprising as a previous report by Liao et al.^33^ clearly demonstrated that the abundance of crRNAs in a CRISPR array varies widely. The variation in the abundance of crRNAs is very likely to be due to the effect of secondary structures which inhibit the formation of the characteristic hairpin required for Cas12a processing^18,33^. To assess this possibility, we predicted the secondary structure of the transcribed pre-crRNAs using NUPACK^34^.

As expected, complex secondary structures were formed in all the pre-crRNAs (Fig. S7). In the CRISPR arrays where the spo0A spacer preceded the upp spacer, an undisrupted hairpin was formed between nucleotide 22 and 35 of the pre-crRNA, recommending the successful recognition and processing of the spo0A crRNA by FnCas12a. However, a secondary structure was observed between the nucleotides of the spo0A spacer, although with low equilibrium probability (Fig. S7). The secondary structure formed by the spo0A spacer may reflect the low knockout efficiency observed in all the multiplex editing assays, rendering the spo0A crRNA as a poorly performing crRNA^30^. Yet, 100% knockout efficiency was observed when the spo0A crRNA was used for single *spo0A* knockouts (Fig. 3B). In contrast to the spo0A spacer, the necessary hairpin for processing the upp1 or upp2 crRNA was disturbed in the spo0A-upp1 and spo0A-upp2 arrays. Still, our results for both the spo0A-upp1 and spo0A-upp2 arrays show that the (hypothetically) well processed spo0A crRNA yields low knockout efficiency for the *spo0A* gene, whereas the disturbed upp1 or upp2 crRNAs are not necessarily a limitation for knocking out the *upp* gene (Fig. 6A). When the upp2 spacer preceded the spo0A spacer, disturbed hairpins were observed for both the upp2 and spo0A crRNAs (Fig. S7). In contrast, in the case where the upp1 spacer preceded the spo0A spacer, undisturbed hairpins were formed for both crRNAs. The upp1-spo0A array combination was the only combination that yielded (clean) double knockouts with a knockout efficiency of 18% (Fig. 6B), which is very likely to be the result of undisturbed hairpins.

In total, our multiplex knockout results cannot be fully explained by the pre-crRNA secondary structure. In most cases, the spo0A crRNA hairpin is structured but yields very low knockout efficiency, whereas the upp crRNA hairpin is unstructured and often yields high knockout efficiency. Based on our observations and the observations made by Liao et al.^33^ and Creutzburg et al. (2020), we can conclude that when a multiplex approach is considered, the test of multiple spacers targeting the same gene in combination with the change in spacer position in the CRISPR array should be applied for optimal results.

## Conclusion

In this study, we successfully developed a CRISPR-FnCas12a genome engineering tool for *C. beijerinckii* NCIMB 8052 that can facilitate single- and multi-plex gene knockout in a single step. The knockout efficiency for single genes varied between 25 and 100%, indicating that different genomic loci are not targeted and deleted equally. The knockout efficiency for the simultaneous deletion of two genes was 18% and dependent on the spacer sequence and position. In general, our tool expands the CRISPR-Cas toolbox in *Clostridia* species and can contribute to the rapid and easy generation of mutants.

## Materials and methods

### Microbial strains and growth conditions

Table 1 shows all the strains used or generated in this study. *Escherichia coli* NEB® 5-alpha was used for plasmid assembly and cloning following the manufacturer’s instructions (New England Biolabs Inc.). Transformed *E. coli* cells were grown at 37 °C in LB liquid medium (10 g L^−1^ tryptone, 5 g L^−1^ yeast extract, 10 g L^−1^ NaCl) or on LB agar plates (LB liquid medium, 15 g L^−1^ bacteriological agar) containing spectinomycin (0.1 g L^−1^).

Transformed *C. beijerinckii* NCIMB 8052 cells were grown anaerobically at 37 °C in modified clostridial growth medium containing glucose as the main carbon source (mCGM-G: 5 g L^−1^ yeast extract, 0.75 g L^−1^ KH_2_PO_4_, 0.75 g L^−1^ K_2_HPO_4_, 0.4 g L^−1^ MgSO_4_. 7H_2_O, 0.01 g L^−1^ MnSO_4_. H_2_O, 0.01 g L^−1^ FeSO_4_. 7H_2_O, 1 g L^−1^ NaCl, 2 g L^−1^ L-asparagine, 2 g L^−1^ (NH_4_)_2_SO_4_, 0.125 g L^−1^ l-cysteine, 13.753 g L^−1^ D-(+)-glucose. H_2_O) or on mCGM-G agar (1 g L^−1^ yeast extract, 2 g L^−1^ tryptone, 0.5 g L^−1^ KH_2_PO_4_, 1 g L^−1^ K_2_HPO_4_, 0.1 g L^−1^ MgSO_4_. 7 H_2_O, 0.01 g L^−1^ MnSO_4_. H_2_O, 0.015 g L^−1^ FeSO_4_. 7 H_2_O, 0.01 g L^−1^ CaCl_2_, 0.002 g L^−1^ CoCl_2_, 0.002 g L^−1^ ZnSO_4_, 2 g L^−1^ (NH_4_)_2_SO_4_, 55 g L^−1^ D-(+)-glucose. H_2_O, 12 g L^−1^ agar) supplemented with 0.65 g L^−1^ spectinomycin.

For fermentation assays and knockout generation, *C. beijerinckii* NCIMB 8052 cells were grown in GAPES medium (2.5 g L^−1^ yeast extract, 1 g L^−1^ KH_2_PO_4_, 0.61 g L^−1^ K_2_HPO_4_, 1 g L^−1^ MgSO_4_ . 7H_2_O, 0.0066 g L^−1^ FeSO_4_. 7 H_2_O, 2.9 g L^−1^ C_2_H_7_NO_2_, 0.19 g L^−1^ pABA, 0.125 g L^−1^ l-cysteine, 65.6 g L^−1^ D-(+)-glucose. H_2_O) supplemented with 0.65 g L^−1^ spectinomycin^35^.

To induce the expression of *FnCas12a*, transformed *C. beijerinckii* NCIMB 8052 cells were plated on mCGM-X agar (containing 40 g L^−1^ xylose instead of glucose as the carbon source) supplemented with 0.65 g L^−1^ spectinomycin.

### Plasmid construction and transformation

The plasmids used in this study are shown in Table 2. Unless otherwise specified, all plasmids were assembled through NEBuilder^®^ HiFi DNA Assembly (NEB). The basic backbone plasmid pCOMA_NT-crRNA was constructed by amplifying the pCB102 ori, colE1 ori and aad9 from pWUR100S (pS), FnCas12a from pY002 and the XylR-XylBP from pE_X_cas9. The crRNA was ordered as synthetic gene fragment (Twist Bioscience).

**Table 2:** Plasmids used in this study.

Plasmid name	Relevant characteristics	Reference	Benchling link
pY002	p15A ori, Lacp-WT FnCas12a, TetR, CmR	^18^	https://benchling.com/s/seq-YBcl7eaFC7NKYg6E2LdA?m=slm-vYGnLxCvJAGubNbYmE65
pWUR100S (pS)	pCB102 ori, colE1 ori, Aad9R	^39^	https://benchling.com/s/seq-IF3obXwGT4j2FO6hQMMY?m=slm-oOjBKIOoNEz6PQAyEWLh
pE_X_cas9	colE1 ori, pAMβ1 ori, 2 μ ori, AmpR, ErmR, URA3	^39^	N/A
pCOMA_NT-crRNA	pCB102 ori, colE1 ori, ThlP-NT-crRNA-ThlT, XylRP-XylR, XylBP-FnCas12a-FdxT, Aad9R	This study	https://benchling.com/s/seq-kRZphFwSRG7C32eUfIaM?m=slm-Rd1jRxe8i8U95StZASKt
pCOMA_spo0A-crRNA	pCB102 ori, colE1 ori, ThlP-spo0A-crRNA-ThlT, XylRP-XylR, XylBP-FnCas12a-FdxT, Aad9R	This study	https://benchling.com/s/seq-RvGvxram3fP7WQB7TmQv?m=slm-Km6u4qNeZy0tqR0vjoLl
pCOMA_NT-crRNA_spo0AHA	pCB102 ori, colE1 ori, ThlP-NT-crRNA-ThlT, XylRP-XylR, XylBP-FnCas12a-FdxT, Aad9R, 500 bp homologous arms for spo0A	This study	https://benchling.com/s/seq-k0Se57aU5vU5yJtJJ9EA?m=slm-BMCrlwYKLJFdUeQE44w8
pCOMA_spo0A-crRNA_spo0AHA	pCB102 ori, colE1 ori, ThlP-spo0A-crRNA-ThlT, XylRP-XylR, XylBP-FnCas12a-FdxT, Aad9R, 500 bp homologous arms for spo0A	This study	https://benchling.com/s/seq-Q3JvVFC0K4ryyyYtCucq?m=slm-lVFdreUu9pHFiRSj1egj
pCOMA_NT-crRNA_uppHA	pCB102 ori, colE1 ori, ThlP-NT-crRNA-ThlT, XylRP-XylR, XylBP-FnCas12a-FdxT, Aad9R, 500 bp homologous arms for upp	This study	https://benchling.com/s/seq-KZXvQiiIsggjBmgPx0PM?m=slm-PhuUqI9ZLVwgnhFylQwn
pCOMA_upp1-crRNA_uppHA	pCB102 ori, colE1 ori, ThlP-upp1-crRNA-ThlT, XylRP-XylR, XylBP-FnCas12a-FdxT, Aad9R, 500 bp homologous arms for upp	This study	https://benchling.com/s/seq-CXSQpeQ1P8z3gpOEEWft?m=slm-dkWwTgCocniSrTiGyuKn
pCOMA_upp2-crRNA_uppHA	pCB102 ori, colE1 ori, ThlP-upp2-crRNA-ThlT, XylRP-XylR, XylBP-FnCas12a-FdxT, Aad9R, 500 bp homologous arms for upp	This study	https://benchling.com/s/seq-tzOIKIu3Jvem9EUyjS73?m=slm-4GndpMy7juE5MjKfB1iq
pCOMA_Cbei_1291-crRNA_Cbei_1291HA	pCB102 ori, colE1 ori, ThlP- Cbei_1291-crRNA-ThlT, XylRP-XylR, XylBP-FnCas12a-FdxT, Aad9R, 500 bp homologous arms for Cbei_1291	This study	https://benchling.com/s/seq-r9BuYMhm43BKMaUACoOj?m=slm-cYcyglDUo8CUuTlYe2ru
pCOMA_ Cbei_3238-crRNA_Cbei_3238HA	pCB102 ori, colE1 ori, ThlP- Cbei_3238-crRNA-ThlT, XylRP-XylR, XylBP-FnCas12a-FdxT, Aad9R, 500 bp homologous arms for Cbei_3238	This study	https://benchling.com/s/seq-EOsV2CMq8B6jPKh6FaGw?m=slm-HHlN6uVjWeIysiygOGXJ
pCOMA_ Cbei_3932-crRNA_Cbei_3932HA	pCB102 ori, colE1 ori, ThlP- Cbei_3932-crRNA-ThlT, XylRP-XylR, XylBP-FnCas12a-FdxT, Aad9R, 500 bp homologous arms for Cbei_3932	This study	https://benchling.com/s/seq-vL6jyhBKUitQN6c5pnxf?m=slm-6X7iV1qYw5dqjETqQ1dP
pCOMA_NT-crRNA_spo0AHA_uppHA	pCB102 ori, colE1 ori, ThlP-NT-crRNA-ThlT, XylRP-XylR, XylBP-FnCas12a-FdxT, Aad9R, 500 bp homologous arms for spo0A, 500 bp homologous arms for upp	This study	https://benchling.com/s/seq-I86Rb364esyrIkKi9VLb?m=slm-oxstw9iRvC8DLpN70viW
pCOMA_spo0A-upp1-crRNA_spo0AHA_uppHA	pCB102 ori, colE1 ori, ThlP-spo0A-upp1-crRNA-ThlT, XylRP-XylR, XylBP-FnCas12a-FdxT, Aad9R, 500 bp homologous arms for spo0A, 500 bp homologous arms for upp	This study	https://benchling.com/s/seq-Wu11w1Ts2vDji2swudwZ?m=slm-hxse2mhHNuvV53hmvIqK
pCOMA_upp1-spo0A-crRNA_spo0AHA_uppHA	pCB102 ori, colE1 ori, ThlP-upp1-spo0A-crRNA-ThlT, XylRP-XylR, XylBP-FnCas12a-FdxT, Aad9R, 500 bp homologous arms for spo0A, 500 bp homologous arms for upp	This study	https://benchling.com/s/seq-mA2bZRiPk9eh1AeBBttp?m=slm-1x0eBFVfYSVSGJRUqp87
pCOMA_spo0A-upp2-crRNA_spo0AHA_uppHA	pCB102 ori, colE1 ori, ThlP-spo0A-upp2-crRNA-ThlT, XylRP-XylR, XylBP-FnCas12a-FdxT, Aad9R, 500 bp homologous arms for spo0A, 500 bp homologous arms for upp	This study	https://benchling.com/s/seq-TnseLEE2T1AkBFi7bWL3?m=slm-KF9mZ2ogPrDjDBjEq9Vq
pCOMA_upp2-spo0A-crRNA_spo0AHA_uppHA	pCB102 ori, colE1 ori, ThlP-upp2-spo0A-crRNA-ThlT, XylRP-XylR, XylBP-FnCas12a-FdxT, Aad9R, 500 bp homologous arms for spo0A, 500 bp homologous arms for upp	This study	https://benchling.com/s/seq-01vSOAWosz7uiyTBz8PH?m=slm-ASg5eXDRVCBE6YGaFyuj

To introduce the homology arms into the pCOMA_NT-crRNA plasmid series, 1 μg pCOMA_NT-crRNA was linearized using AccI (NEB). The linear backbone was then dephosphorylated using shrimp alkaline phosphatase (rSAP, NEB) following the manufacturer’s instructions. rSAP and residual AccI were deactivated by incubating the solution at 80 °C for 20 min. The linearized backbone was further purified using the DNA clean and concentrator kit (Zymo Research). The homology arms were amplified by PCR using *C. beijerinckii* NCIMB 8052 genomic DNA as template and the oligonucleotides listed in Table S5. The correct size of the homology arms was confirmed through gel electrophoresis. Following, the amplicons were gel purified using the zymoclean gel DNA recovery kit (Zymo Research) and introduced into the linearized pCOMA_NT-crRNA through NEBuilder^®^ HiFi DNA Assembly (NEB), following the instructions from the manufacturer. 5 μL of the assembly was used to transform *E. coli* NEB^®^ 5-alpha cells (NEB). Transformed cells were plated on LB agar containing spectinomycin (0.1 g L^−1^) and incubated at 37 °C overnight. Obtained colonies were screened through PCR and the obtained plasmids were sequenced for the correctness of the homology arm insert using Sanger sequencing (Macrogen Europe B.V.; Table 2).

Single or double targeting spacers were introduced through Golden Gate assembly using an adapted protocol^37^. Briefly, 1 μL of each of the two complementary oligonucleotides (100 μM each; Table S5) were mixed with 1 μL NaCl (1 M) and 47 μL MQ water and incubated at 95 °C for 5 min. Following, the solution was cooled down at room temperature for at least 2 h to achieve annealing of the complementary oligonucleotides. The annealed oligonucleotides were then diluted 10 times and 2 μL of the diluted oligonucleotides was mixed with 2 μL (0.01–0.02 pmol μL^−1^) of the appropriate pCOMA_NT-crRNA plasmid series and 2 μl of MetaMix stock (10 μl BsaI-HF^®^v2, 15 μL T4 ligation buffer, 10 μL T4 ligase and 15 μL MQ). The mix solution was then incubated in a thermocycler using the following protocol: 5 min at 37 °C, 5 min at 16 °C followed by 5 min at 37 °C (repeat for 15–30 cycles), 5 min at 37 °C, 20 min at 80 °C. 1 μL of the solution was then used to transform chemically competent *E. coli* NEB^®^ 5-alpha (NEB) according to the instructions of the manufacturer. Transformed cells were plated on LB agar plates containing spectinomycin (0.1 g L^−1^) and incubated overnight at 37 °C. Obtained colonies were used for plasmid cloning by growing them in 10 mL LB medium containing spectinomycin (0.1 g L^−1^) and incubating overnight at 37 °C. Plasmid purification was performed by using the GeneJET Plasmid Miniprep kit (ThermoFischer Scientific) and following the manufacturer’s instructions. To verify the spacer(s) sequence, plasmids were sequenced using Sanger sequencing (Macrogen Europe B.V.; Table 2).

*Clostridium* *beijerinckii* was transformed as previously described^38^. In detail, 100 μL of heat-shocked *C. beijerinckii* spores (1 min at 99 °C) were used to inoculate 25 mL of mCGM liquid medium followed by overnight incubation at 37 °C. 20 mL of the overnight culture was used to inoculate 180 mL of pre-warmed (37 °C) mCGM liquid medium which was then incubated at 37 °C until an OD_600_ equal to 0.3–0.4 was reached. Following, in an anaerobic tent, the culture was transferred into a 400-mL sterile centrifuge tube which was then sealed with parafilm to limit oxygen entrance. The culture was then centrifuged aerobically at 6000 rpm at 4 °C for 10 min. The centrifuged culture was put on ice and transferred again in the anaerobic tent. The supernatant was then discarded and the cell pellet was resuspended with 25 mL of ice-cold anaerobic electroporation buffer (270 mM D-sucrose, 1 mM sodium phosphate buffer pH 7.4, 1 mM MgCl_2_). The resuspended culture was transferred into a 30-mL sterile centrifuge tube which was then sealed with parafilm to limit oxygen entrance. The resuspended culture was then centrifuged aerobically at 6000 rpm at 4 °C for 10 min followed by discarding the supernatant and resuspending the cell pellet with 1.5 mL of ice-cold anaerobic electroporation buffer. 300 μL of the resuspended cells were used to electroporate (1.25 kV, 25 μF, 100 D) 3–5 μg plasmid DNA using 0.2 cm ice-cold electroporation cuvettes. After electroporation, the transformants were recovered at 37 °C in 3 mL of anaerobic mCGM for 3 h. After recovery, cultures were centrifuged at 6000 rpm for 5 min and the cell pellet was plated on mCGM solid medium containing spectinomycin (0.65 g L^−1^) followed by incubation at 37 °C for 72–96 h. Obtained colonies were screened for the presence of FnCas12a, the crRNA and the homology arms through colony PCR. Correct transformants were subcultured in mCGM and stored as vegetative cells in 20% glycerol at − 80 °C until use.

### CRISPR-Cas12a spacer selection

The selection of Cas12a CRISPR spacers is based on the knowledge obtained from Zetsche et al.^18^, Hui et al.^30^, Creutzburg et al.^31^ and Liao et al.^33^. To find spacers in the target genomic locus, the CRISPR Guide (guide is used interchangeably with spacer) RNA design tool developed by Benchling was used (Benchling Inc.). Firstly, a 20 bp single guide with a custom 5′-TTV-3′ PAM at the 5′ site of the protospacer was used as selection parameter. Since the genome of *C. beijerinckii* NCIMB 8052 was not available on the Benchling drop down menu, we selected the closest available *Clostridium* genome (*Clostridium saccharobutylicum* DSM 13,864) defined by using the genome clustering tool provided by the MicroScope platform (Genoscope). Following, the target genomic locus was used to screen for Cas12a guides, resulting in a list of possible guides. From the obtained list, we exluded guides that started with a thymine as it was previously described to be inefficient^31^. Then, we screened for potential off-target effects by aligning the first ten nucleotides of the guides to the genome of the target organism and searched for the presence of a 5′-TTV-3′ PAM at the 5′ site of the protospacer. After selecting guides with only one possible target location (i.e. the target site), we screened the guides for complex secondary structures that inhibit the loop formation in the repeat sequence of the CRISPR-Cas12a crRNA^30,33^. To perform such analysis, we used the complete crRNA transcript sequence as expressed from our plasmids (repeat-spacer-repeat) and used it to predict the RNA secondary structure in online tools such as mFold (www.unafold.org) or NUPACK (www.nupack.org). Guides which did not show complex structures that inhibited the loop formation of the CRISPR-Cas12a crRNA were finally selected and cloned in our plasmid vectors as described above.

### *C. beijerinckii* NCIMB 8052 knockout generation

*Clostridium* *beijerinckii* cells transformed with the pCOMA plasmid series were grown in 25 mL selective GAPES medium for 48–96 h to allow for homologous recombination to occur. 100 μL of the fully grown culture was then plated on selective mCGM-X agar plates to induce expression of *FnCas12a* and allow for the counterselection of the mutants. Agar plates were incubated for 24–48 h at 37 °C and obtained colonies were screened through colony PCR. To perform colony PCR, colonies were resuspended in 50 μL PBS buffer (pH 7.4) and heated for 10 min at 99 °C. The solution was then centrifuged and 1 μL of the supernatant was used as template for PCR using the Q5^®^ High-Fidelity DNA Polymerase (NEB), following the manufacturer’s instructions. The primers used for colony PCR are listed in Table S5. Lastly, to confirm the complete knockout of the gene of interest, PCR amplicons were sequenced through Sanger sequencing (Macrogen Europe B.V.) and the result can be found in the associated benchling link in Table 1 and Supplementary Table 6. Each knockout experiment was performed in triplicate and the average knockout efficiency was calculated by defining the percentage of clean mutants (i.e., no mixed bands) versus wild type and mix bands.

### Plasmid curing

To cure the *C. beijerinckii* NCIMB 8052 knockout strains of the pCOMA plasmids, the cells were grown in 25 mL mCGM-G liquid medium without antibiotics for 24 h. 100 μL of the grown culture was then plated on mCGM-G agar plates without the presence of antibiotics and grown for 24 h at 37 °C. Obtained colonies were randomly selected and streaked out on one mCGM-G agar plate with antibiotics and on one mCGM-G agar plate without antibiotics and incubated for 24 h at 37 °C. Colonies that did not grow on selective medium but grew on non-selective medium were selected and screened for the absence of plasmid through colony PCR. Mutant colonies which lost the respective pCOMA plasmid were grown in GAPES medium without antibiotics and glycerol stocks were made and stored at − 80 °C until further use.

### Δ*spo0A* and WT *C. beijerinckii* NCIMB 8052 fermentation assays and morphology

The WT and Δ*spo0A C. beijerinckii* NCIMB 8052 strains were grown in GAPES medium without antibiotics for 48 h. At different time intervals, 1 mL of headspace was recovered and the solvent concentration was determined using gas chromatography (GC). 1 mL of liquid culture was also recovered, of which the pH, OD_600_ and organic acid concentration were determined using high-pressure liquid chromatography (HPLC).

A Shimadzu GC-2010 equipped with an Agilent technologies DB-WAX UI GC column (30 m × 0.53 mm) using a temperature gradient of 60–125 °C over 10 min and a nitrogen flow rate of 115 mL min^−1^ was used to separate metabolites. A split ratio of 20 and a carrier flow program with a constant pressure of 30 kPa was applied. References of GAPES medium containing 100, 50, 20 and 5 mM of acetone, ethanol and butanol was used to create a calibration curve. As internal standard, 5 mM of 1-propanol was used.

For HPLC, a Shimadzu LC-2030 with a Shimadzu RID-20A detector was used. To separate the metabolites, a Shodex SUGAR SH1821 column was operated at 45 °C with a flow rate of 0.8 mL min^−1^ and a flow time of 20 min. 0.01N H_2_SO_4_ was used as eluent. References of 100; 50; 25; 12.5; 6.25; 3.125, 1.5625 and 0.78125 mM of lactate, acetate and butyrate were used to create a calibration curve. As internal standard, 5 mM of crotonate was used.

Pictures of WT and Δ*spo0A C. beijerinckii* NCIMB 8052 colonies were taken on mCGM agar plates after 48 h of incubation at 37 °C, using Carl Zeiss Axio Scope.A1, 100× total magnification, phase 1.

## Supplementary Information

Supplementary Information.

## Data Availability

PCR amplicons sequenced through Sanger sequencing (Macrogen Europe B.V.) can be found in the associated benchling link in Tables 1 and 2 and the raw data supporting the conclusions of this article will be made available by the corresponding author, without undue reservation.
