# Supplementary material for: Multiplex genome engineering in Clostridium beijerinckii NCIMB 8052 using CRISPR-Cas12a
Source: Sci Rep. 2023 Jun 22;13:10153. doi: 10.1038/s41598-023-37220-y (PMC10287719; doi:10.1038/s41598-023-37220-y)
Supplement: Supplementary file 1 — Supplementary Information. [file 41598_2023_37220_MOESM1_ESM.docx]

Multiplex genome engineering in *Clostridium beijerinckii* NCIMB 8052 using CRISPR-Cas12a

Constantinos Patinios^1,2,a^, Stijn T. de Vries^1,a^, Mamou Diallo^1,3,a^, Lucrezia Lanza^1^, Pepijn L. J. V. Q. Verbrugge^1^, Ana M. López-Contreras^3^, John van der Oost^1^, Ruud A. Weusthuis^2,^ Servé W. M. Kengen^1*^

^1^Laboratory of Microbiology, Wageningen University and Research, Stippeneng 4, 6708 WE Wageningen, the Netherlands.

^2^Bioprocess Engineering, Wageningen University and Research, Droevendaalsesteeg 1, 6708 PB Wageningen, the Netherlands.

^3^Bioconversion Group, Wageningen Food and Biobased Research, Bornse Weilanden 9, 6708WG Wageningen, the Netherlands

^a^These authors have contributed equally to the work

*Corresponding author: Servé W. M. Kengen (serve.kengen@wur.nl)

# Supplementary information


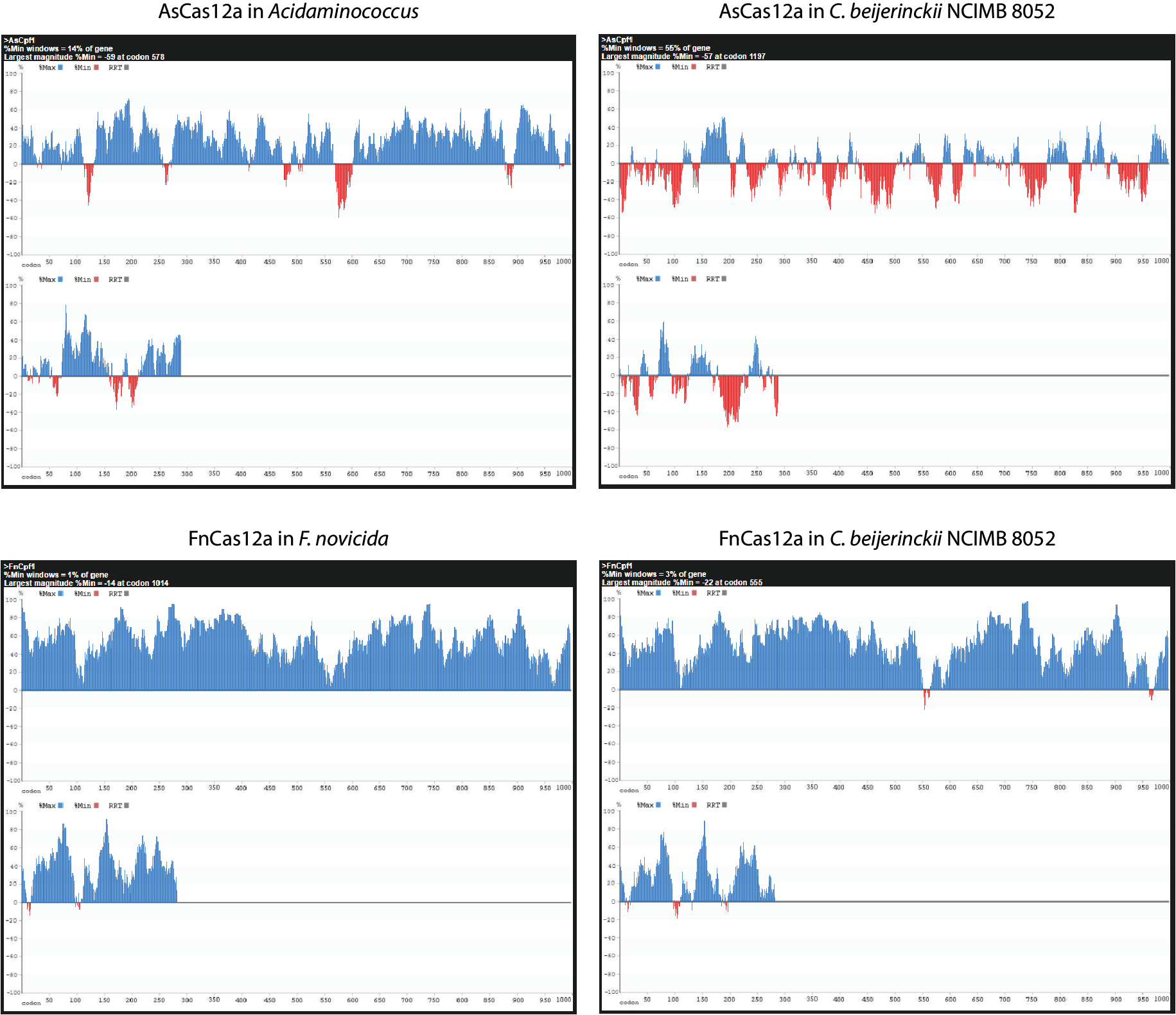


**Supplementary Figure 1. Codon usage of *AsCas12a* and *FnCas12a* in the native (*Acidaminococcus* or *Francisella novicida*, respectively) and target (*C. beijerinckii* NCIMB 8052) organisms.** The codon usage for each organism was created using the codon harmonization tool developed by Claassens et al. (2017). The Cas12a codon usage in the native and target organisms was visualized using the codons.org website created by Clarke T.F. & Clark P.L. (2008).


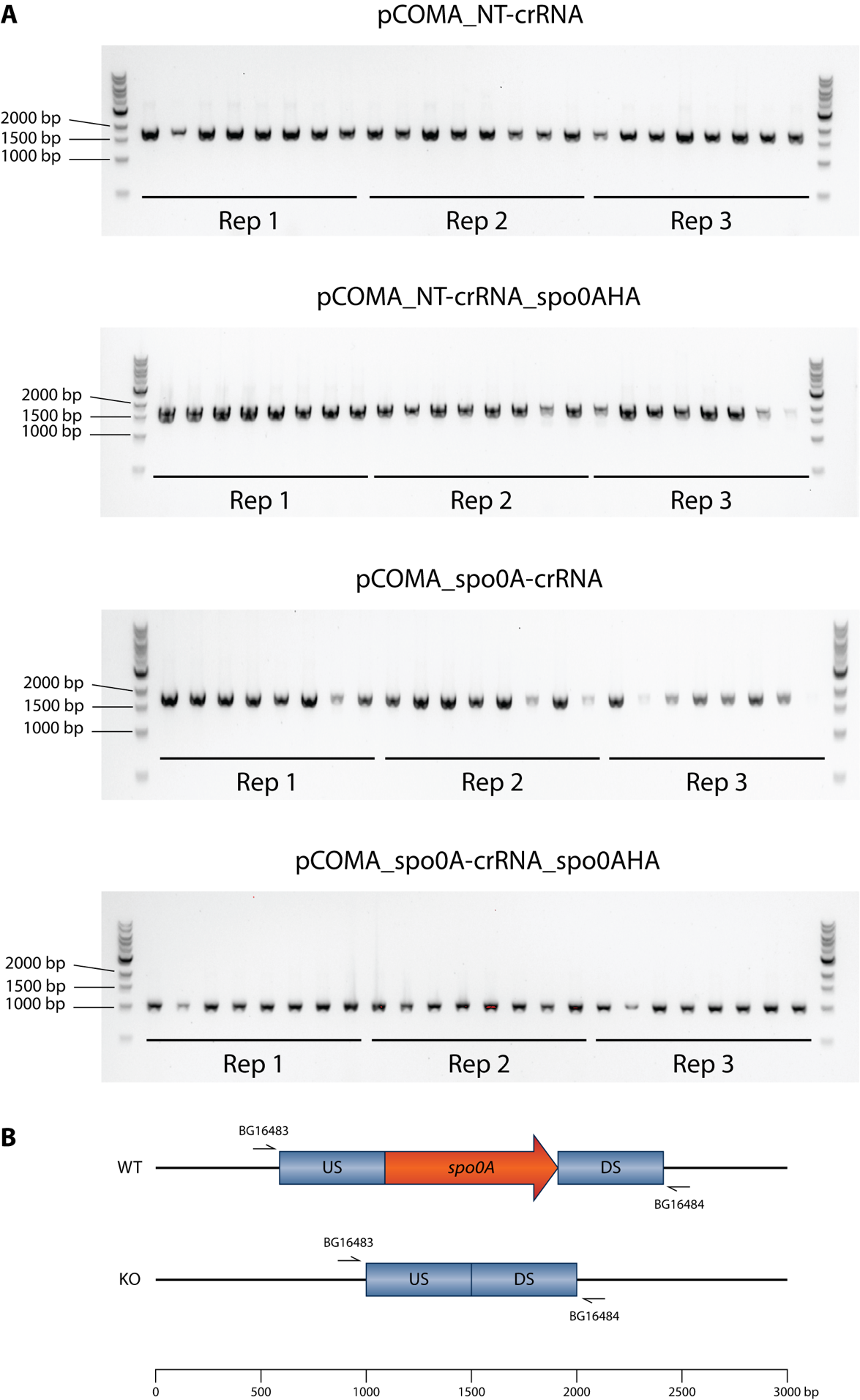


**Supplementary Figure 2. Raw data for the single-gene knockout of *spo0A* using CRISPR-FnCas12a in *C. beijerinckii* NCIMB 8052.** A) *C. beijerinckii* NCIMB 8052 was transformed either with pCOMA_NT-crRNA, pCOMA_NT-crRNA_spo0AHA, pCOMA_spo0A-crRNA or pCOMA_spo0A-crRNA_spo0AHA and obtained colonies were screened through colony PCR using BG16483 and BG16484 oligos (Table S5). This experiment was performed in biological triplicates and the result of each triplicate is represented by Rep1, Rep2 and Rep3 at the bottom of each gel. Wild-type *spo0A*: 1866 bp, Δ*spo0A*: 1044 bp. B) Schematic representation of wild type (WT) or knockout (KO) *C. beijerinckii* NCIMB 8052 genome at the spo0A locus. BG16483 and BG16484 represent the primers used for colony PCR (Table S5). US and DS represent the 500 bp homology arms upstream and downstream of the *spo0A* gene, respectively.


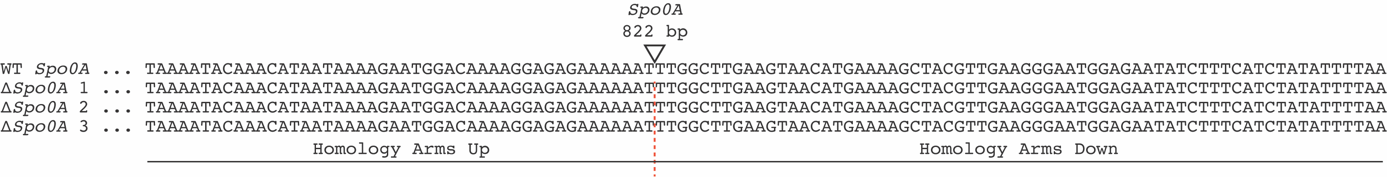


**Supplementary Figure 3. Sequencing results of three Δ*spoA0* isolates.** WT *spo0A* represents the wild type reference genomic locus of *C. beijerinckii* NCIMB 8052 whereas Δ*spoA0* 1, 2 and 3 represent the isolated mutants. The homology arms up and down of the three mutant sequences are adjoined, indicating complete deletion of the 822 bp *spo0A* gene.


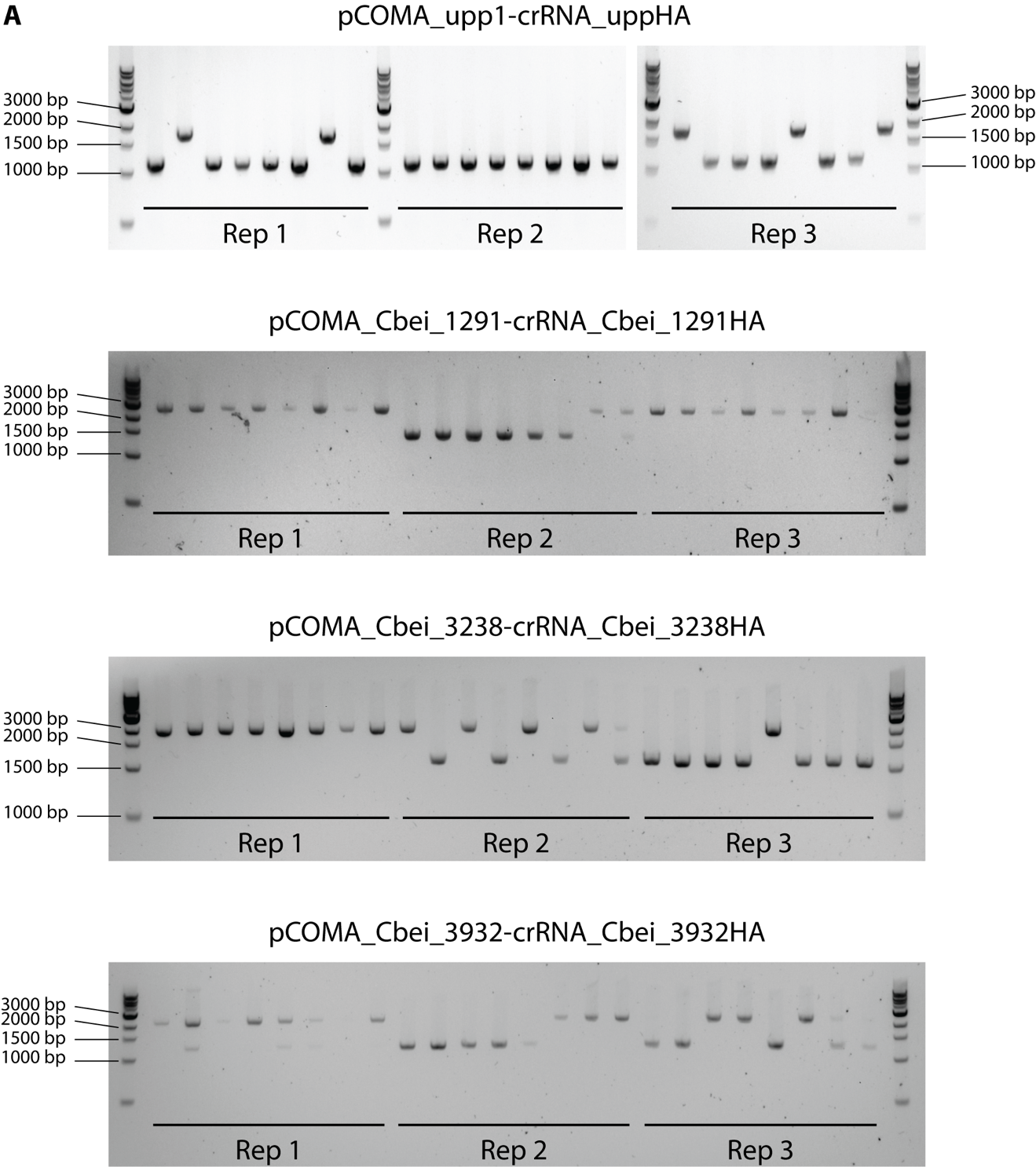


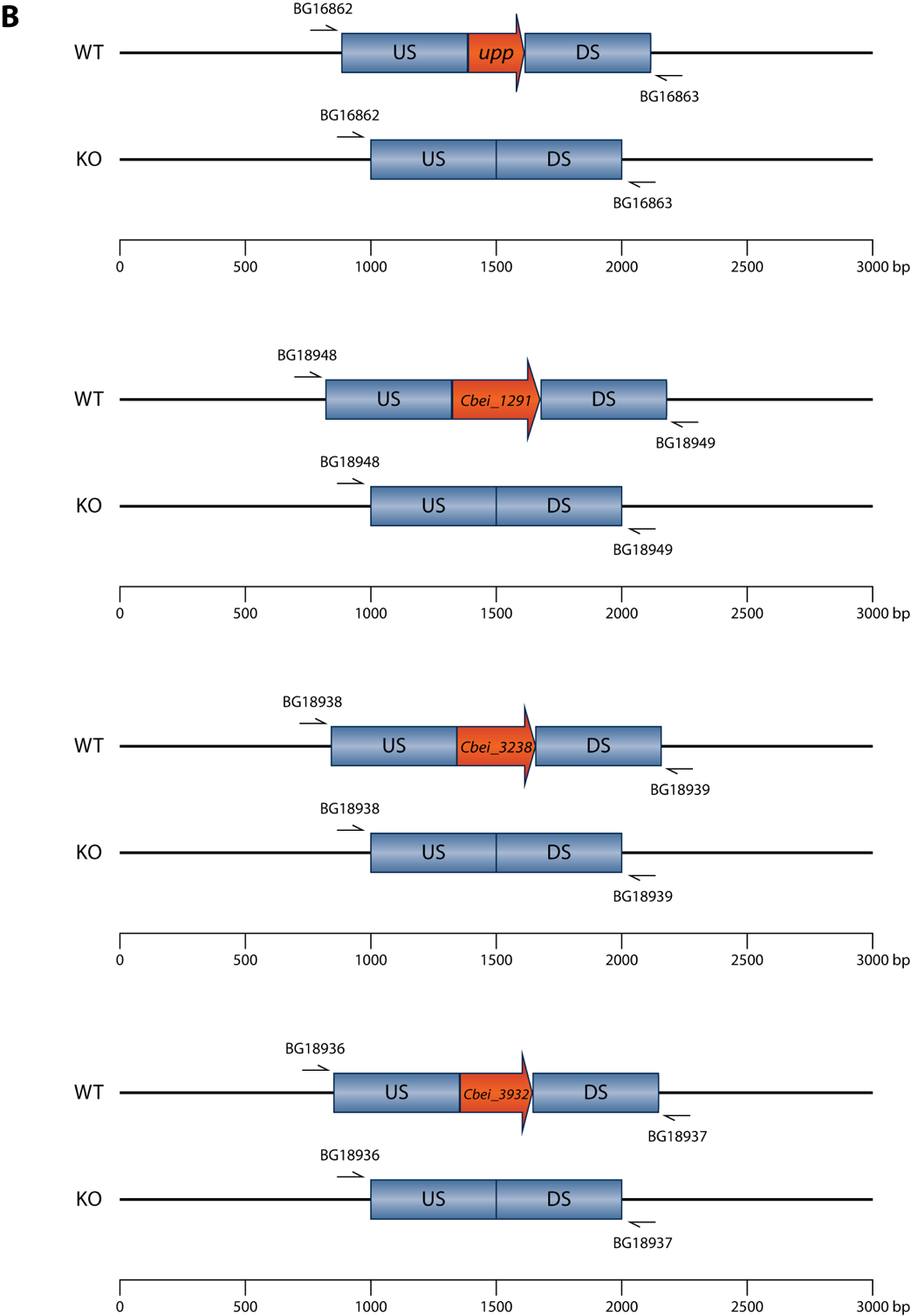


**Supplementary Figure 4. Raw data for the single-gene knockout of the *upp*, *Cbei_1291*, *Cbei_3238* and *Cbei_3932* genes using CRISPR-FnCas12a in *C. beijerinckii* NCIMB 8052.** (**A**) *C. beijerinckii* NCIMB 8052 was transformed with either of pCOMA_upp1-crRNA_uppHA, pCOMA_Cbei_1291-crRNA_Cbei_1291HA, pCOMA_Cbei_3238-crRNA_ Cbei_3238HA or pCOMA_Cbei_3932-crRNA_Cbei_3932HA. Each knockout experiment was performed in biological triplicates and the result of each triplicate is represented by Rep1, Rep2 and Rep3 at the bottom of each gel. Mix amplicons (wild-type and knockout bands) were not counted for the total knockout efficiency percentage. The primers used for this experiment are listed in Table S5. Wild-type *upp*: 1775 bp, Δ*upp*: 1145 bp. Wild-type *Cbei_1291*: 2392 bp, Δ*Cbei_1291*: 1405 bp. Wild-type *Cbei_3238*: 1979 bp, Δ*Cbei_3238*: 1091 bp. Wild-type *Cbei_3932*: 2054 bp, Δ*Cbei_3932*: 1241 bp. (**B**) Schematic representation of wild type (WT) or knockout (KO) *C. beijerinckii* NCIMB 8052 genome at the *upp*, Cbei_1291, Cbei_3238, Cbei_3932 loci. Primers BG16862, BG16863, BG18948, BG18949, BG18938, BG18939, BG18936 and BG18937 represent the primers used for colony PCR (Table S5). US and DS represent the 500 bp homology arms upstream and downstream of the relevant gene, respectively.


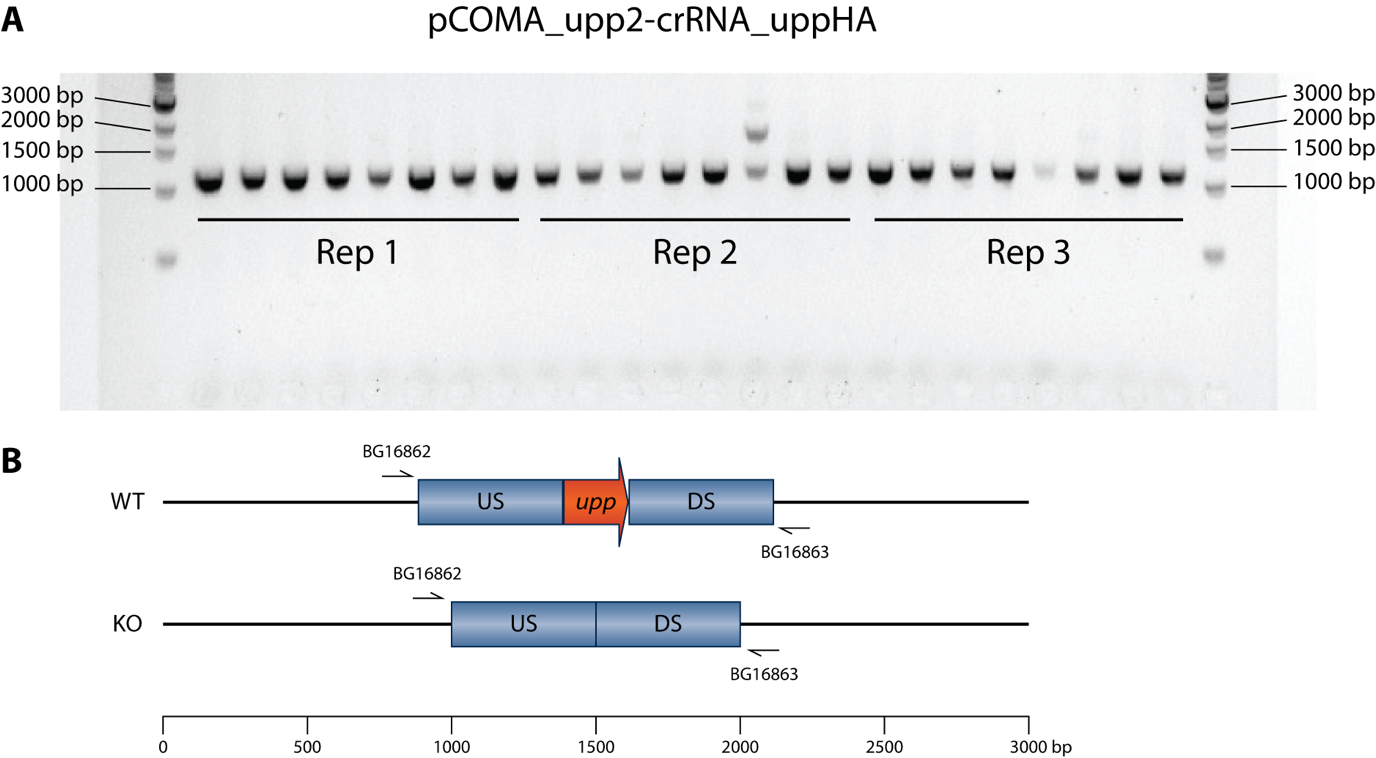


**Supplementary Figure 5. Raw data for the single-gene knockout of the *upp* gene using pCOMA_upp2-crRNA_uppHA.** (**A**) *C. beijerinckii* NCIMB 8052 was transformed with pCOMA_upp2-crRNA_uppHA and obtained colonies were screened for the knockout of the *upp* gene. Each knockout experiment was performed in biological triplicates and the result of each triplicate is represented by Rep1, Rep2 and Rep3 at the bottom of each gel. The primers used for this experiment are listed in Table S5. Wild-type *upp*: 1775 bp, Δ*upp*: 1145 bp. (**B**) Schematic representation of wild type (WT) or knockout (KO) *C. beijerinckii* NCIMB 8052 genome at the *upp* locus. Primers BG16862 and BG16863 represent the primers used for colony PCR (Table S5). US and DS represent the 500 bp homology arms upstream and downstream of the *upp* gene, respectively.


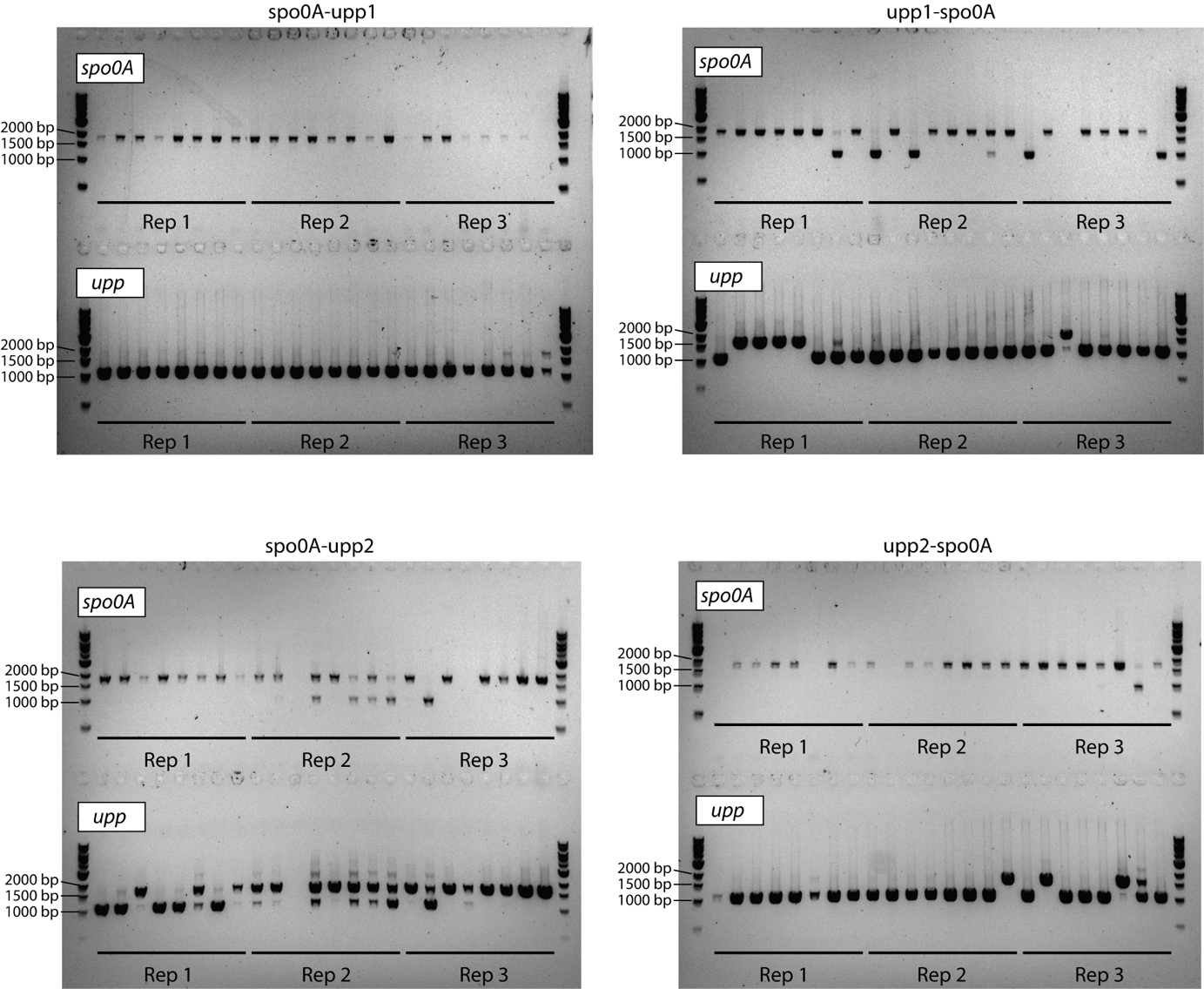


**Supplementary Figure 6. Raw data for the multiplex knockout of *spo0A* and *Upp* using CRISPR-FnCas12a in *C. beijerinckii* NCIMB 8052.** pCOMA_spo0A-upp1-crRNA_spo0AHA_uppHA (top left), pCOMA_upp1-spo0A-crRNA_spo0AHA_uppHA (top right), pCOMA_spo0A-upp2-crRNA_spo0AHA_uppHA (bottom left) or pCOMA_upp2-spo0A-crRNA_spo0AHA_uppHA (bottom right) was used to transform *C. beijerinckii* NCIMB 8052. Each knockout experiment was performed in biological triplicates and the result of each triplicate is represented by Rep1, Rep2 and Rep3 at the bottom of each gel and separated by the dashed vertical lines. Mix amplicons (wild-type and knockout bands) were not counted for the total knockout efficiency percentage. The primers used for this experiment are listed in Table S5. Wild-type *spo0A*: 1866 bp, Δ*spo0A*: 1044 bp. Wild-type *upp*: 1775 bp, Δ*upp*: 1145 bp.


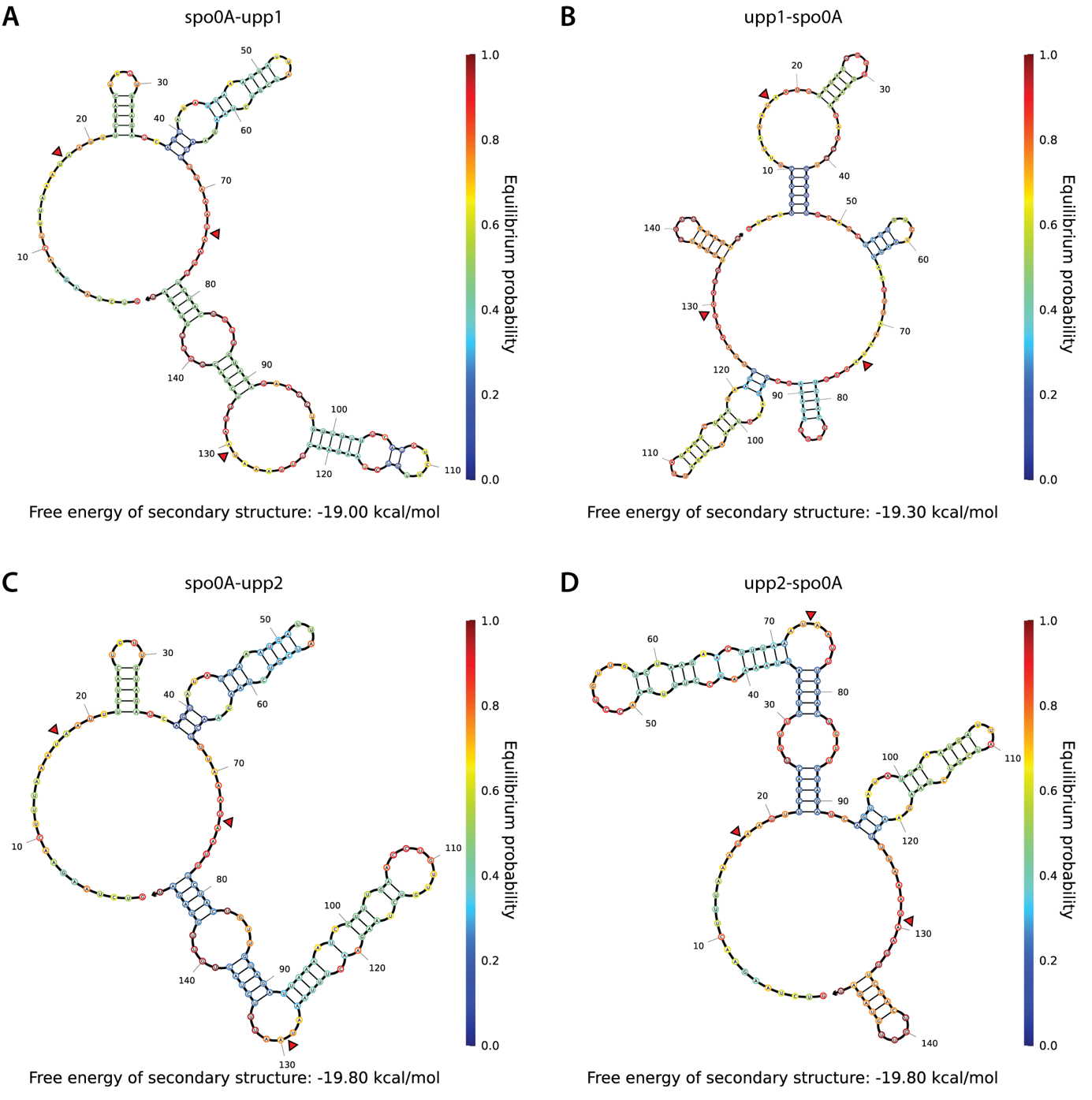


**Supplementary Figure 7. Predicted secondary structure of pre-crRNAs.** The pre-crRNAs include the three 36 nt repeats interspaced with the designated 20 nt spacers. Red triangles indicate the processing site by FnCas12a. A) pre-crRNA where the spo0A spacer precedes the upp1 spacer. B) pre-crRNA where the upp1 spacer precedes the spo0A spacer. C) pre-crRNA where the spo0A spacer precedes the upp2 spacer. D) pre-crRNA where the upp2 spacer precedes the spo0A spacer.

**Supplementary Table 1. Raw data for acid production of WT and Δ*spo0A C. beijerinckii* NCIMB 8052.**

| **No.** | **Strain** | **Time** | **Injection Name** | **Amount** | **Amount** | **Amount** |
| --- | --- | --- | --- | --- | --- | --- |
|  |  | **Hours** |  | **mM** | **mM** | **mM** |
|  |  |  |  | **Lactate** | **Acetate** | **Butyrate** |
| 1 | n.a. | n.a. | H2O | n.a. | n.a. | n.a. |
| 2 | n.a. | n.a. | ASC 0.78 mM | 0,8506 | 0,6953 | 0,9506 |
| 3 | n.a. | n.a. | ASC 1.5625 mM | 1,6004 | 1,4915 | 1,5894 |
| 4 | n.a. | n.a. | ASC 3.125 mM | 3,1554 | 2,5992 | 3,082 |
| 5 | n.a. | n.a. | ASC 6.25 mM | 6,3352 | 6,344 | 6,4721 |
| 6 | n.a. | n.a. | ASC 12.5 mM | 12,4283 | 12,673 | 12,7804 |
| 7 | n.a. | n.a. | ASC 25 mM | 24,6856 | 24,57 | 24,6523 |
| 8 | n.a. | n.a. | ASC 50 mM | 50,1635 | 51,4927 | 49,3061 |
| 9 | n.a. | n.a. | ASC 100 mM | 99,9984 | 99,3519 | 100,3845 |
| 10 | WT | 0 | 1 | 0,2662 | 34,426 | 0,6882 |
| 11 | WT | 0 | 2 | 0,2588 | 33,7395 | 0,7429 |
| 12 | WT | 0 | 3 | 0,2274 | 35,6623 | 0,6074 |
| 13 | WT | 2 | 4 | 0,2203 | 33,0122 | 0,6679 |
| 14 | WT | 2 | 5 | 0,1639 | 32,6163 | 0,728 |
| 15 | WT | 2 | 6 | 0,2073 | 33,1437 | 0,5626 |
| 16 | WT | 4 | 7 | 0,2157 | 34,2339 | 1,0414 |
| 17 | WT | 4 | 8 | 0,2095 | 35,6656 | 0,9875 |
| 18 | WT | 4 | 9 | 0,1693 | 34,2271 | 1,0139 |
| 19 | WT | 6 | 10 | 0,3228 | 34,8054 | 2,2285 |
| 20 | WT | 6 | 11 | 0,3164 | 34,4436 | 2,1043 |
| 21 | WT | 6 | 12 | 0,2776 | 33,9655 | 1,695 |
| 22 | WT | 8 | 13 | 1,008 | 37,4899 | 5,8505 |
| 23 | WT | 8 | 14 | 0,9947 | 38,2861 | 5,0407 |
| 24 | WT | 8 | 15 | 1,023 | 38,2087 | 5,0726 |
| 25 | WT | 10 | 16 | 2,4812 | 36,5754 | 10,3609 |
| 26 | WT | 10 | 17 | 2,2857 | 37,5053 | 10,5627 |
| 27 | WT | 10 | 18 | 2,213 | 37,5789 | 10,3511 |
| 28 | WT | 12 | 19 | 2,5172 | 33,0916 | 14,9115 |
| 29 | WT | 12 | 20 | 2,606 | 31,0169 | 15,2035 |
| 30 | WT | 12 | 21 | 2,441 | 32,4125 | 14,675 |
| 31 | WT | 13 | 22 | 2,1565 | 27,2526 | 18,6048 |
| 32 | WT | 13 | 23 | 1,8525 | 27,8815 | 18,7645 |
| 33 | WT | 13 | 24 | 2,0668 | 28,3605 | 18,5375 |
| 34 | WT | 24 | 25 | 8,4502 | 9,1051 | 22,9842 |
| 35 | WT | 24 | 26 | 7,9016 | 9,6024 | 23,2909 |
| 36 | WT | 24 | 27 | 8,3749 | 8,8023 | 23,6606 |
| 37 | WT | 30 | 28 | 9,2482 | 7,0151 | 27,5535 |
| 38 | WT | 30 | 29 | 8,6611 | 7,1066 | 26,817 |
| 39 | WT | 30 | 30 | 8,9529 | 6,9595 | 26,6012 |
| 40 | WT | 36 | 31 | 7,6406 | 7,1056 | 29,4398 |
| 41 | WT | 36 | 32 | 7,7785 | 6,7202 | 30,1975 |
| 42 | WT | 36 | 33 | 7,6739 | 6,44 | 29,7294 |
| 43 | WT | 48 | 34 | 4,5394 | 7,8925 | 32,514 |
| 44 | WT | 48 | 35 | 5,241 | 7,7573 | 33,0613 |
| 45 | WT | 48 | 36 | 4,6078 | 6,7569 | 32,9633 |
| 46 | Δspo0A | 0 | 37 | 0,1265 | 34,3282 | 0,5815 |
| 47 | Δspo0A | 0 | 38 | 0,1202 | 34,9191 | 0,5034 |
| 48 | Δspo0A | 0 | 39 | 0,0974 | 34,54 | 0,4594 |
| 49 | Δspo0A | 2 | 40 | 0,0924 | 36,8591 | 0,5364 |
| 50 | Δspo0A | 2 | 41 | 0,0805 | 35,2067 | 0,5811 |
| 51 | Δspo0A | 2 | 42 | 0,1054 | 35,4509 | 0,5807 |
| 52 | Δspo0A | 4 | 43 | 0,1808 | 34,5894 | 0,9882 |
| 53 | Δspo0A | 4 | 44 | 0,1479 | 35,2295 | 0,9261 |
| 54 | Δspo0A | 4 | 45 | 0,1649 | 36,2137 | 0,8935 |
| 55 | Δspo0A | 6 | 46 | 0,9484 | 35,2814 | 2,8414 |
| 56 | Δspo0A | 6 | 47 | 0,715 | 34,357 | 2,4348 |
| 57 | Δspo0A | 6 | 48 | 0,6603 | 36,8738 | 2,4667 |
| 58 | Δspo0A | 8 | 49 | 3,169 | 41,19 | 7,0631 |
| 59 | Δspo0A | 8 | 50 | 2,8367 | 40,4672 | 6,1164 |
| 60 | Δspo0A | 8 | 51 | 2,6132 | 41,2408 | 6,4079 |
| 61 | Δspo0A | 10 | 52 | 5,1276 | 43,7866 | 11,773 |
| 62 | Δspo0A | 10 | 53 | 4,8016 | 43,2056 | 10,985 |
| 63 | Δspo0A | 10 | 54 | 4,665 | 42,4143 | 10,7829 |
| 64 | Δspo0A | 12 | 55 | 5,7428 | 43,9665 | 14,6616 |
| 65 | Δspo0A | 12 | 56 | 5,6909 | 44,6455 | 14,2273 |
| 66 | Δspo0A | 12 | 57 | 5,6943 | 44,39 | 13,9923 |
| 67 | Δspo0A | 13 | 58 | 6,0314 | 44,9779 | 16,2733 |
| 68 | Δspo0A | 13 | 59 | 6,0855 | 45,5951 | 16,2388 |
| 69 | Δspo0A | 13 | 60 | 5,9965 | 44,9693 | 15,7933 |
| 70 | Δspo0A | 24 | 61 | 8,2123 | 46,6459 | 21,617 |
| 71 | Δspo0A | 24 | 62 | 8,3574 | 46,4415 | 20,9786 |
| 72 | Δspo0A | 24 | 63 | 8,5281 | 46,5012 | 20,7735 |
| 73 | Δspo0A | 30 | 64 | 7,9732 | 46,5061 | 21,6264 |
| 74 | Δspo0A | 30 | 65 | 8,2065 | 46,4983 | 20,926 |
| 75 | Δspo0A | 30 | 66 | 8,2501 | 46,339 | 21,0855 |
| 76 | Δspo0A | 36 | 67 | 8,614 | 46,5042 | 21,6097 |
| 77 | Δspo0A | 36 | 68 | 8,479 | 39,5086 | 20,9553 |
| 78 | Δspo0A | 36 | 69 | 8,4227 | 45,7044 | 20,9701 |
| 79 | Δspo0A | 48 | 70 | 8,2082 | 46,5071 | 21,3581 |
| 80 | Δspo0A | 48 | 71 | 8,4418 | 45,4631 | 20,7078 |
| 81 | Δspo0A | 48 | 72 | 8,6994 | 46,1695 | 20,9583 |

ASC=Acids Standard Curve. Acetate, Lactate and Butyrate were used in different concentrations for the Standard Curve. Crotonate was used as Internal Standard. n.a=not applicable

**Supplementary Table 2. Raw data for solvent production of WT and Δ*spo0A C. beijerinckii* NCIMB 8052.**

| **No.** | **Strain** | **Injection Name** | **Time** | **Amount (mM)** | **Amount (mM)** | **Amount (mM)** |
| --- | --- | --- | --- | --- | --- | --- |
|  |  |  | **Hours** | **Acetone** | **Ethanol** | **Butanol** |
| 1 | n.a. | H2O | n.a. | n.a. | n.a. | n.a. |
| 2 | n.a. | SSC 100 mM | n.a. | 99,6615 | 102,6618 | 100,8788 |
| 3 | n.a. | SSC 50 mM | n.a. | 51,9727 | 48,5028 | 50,7904 |
| 4 | n.a. | SSC 20 mM | n.a. | 16,2258 | 10,4859 | 14,0036 |
| 5 | n.a. | SSC 5 mM | n.a. | 7,1401 | 4,7922 | 3,5055 |
| 6 | n.a. | SSC 2.5 mM | n.a. | 2,7493 | 2,6663 | 2,2455 |
| 7 | n.a. | SSC 1 mM | n.a. | 1,5655 | 1,0667 | 1,215 |
| 8 | n.a. | SSC 0.5 mM | n.a. | 0,4129 | 0,5691 | 0,5483 |
| 9 | n.a. | SSC 0.25 mM | n.a. | 0,2276 | 0,2367 | 0,3085 |
| 10 | n.a. | SSC 0.125 mM | n.a. | 0,1511 | 0,0984 | 0,1302 |
| 11 | WT | 1 | 0 | 3,4555 | 0,0356 | 0,2468 |
| 12 | WT | 2 | 0 | 3,4563 | 0,033 | 0,2845 |
| 13 | WT | 3 | 0 | 3,4582 | 0,0375 | 0,2727 |
| 14 | Δspo0A | 4 | 0 | 3,3618 | 0,0096 | 0,0002 |
| 15 | Δspo0A | 5 | 0 | 3,3621 | 0,1029 | 0,0021 |
| 16 | Δspo0A | 6 | 0 | 3,3625 | 0,1166 | 0,0044 |
| 17 | WT | 7 | 2 | 3,5357 | 0,1406 | 0,3098 |
| 18 | WT | 8 | 2 | 3,5154 | 0,1735 | 0,2402 |
| 19 | WT | 9 | 2 | 3,518 | 0,154 | 0,2751 |
| 20 | Δspo0A | 10 | 2 | 3,3616 | 0,1415 | 0,0027 |
| 21 | Δspo0A | 11 | 2 | 3,3623 | 0,0871 | 0,0014 |
| 22 | Δspo0A | 12 | 2 | 3,3627 | 0,1398 | 0,0011 |
| 23 | WT | 13 | 4 | 3,5716 | 0,0879 | 0,2968 |
| 24 | WT | 14 | 4 | 3,5782 | 0,4645 | 0,2341 |
| 25 | WT | 15 | 4 | 3,5915 | 1,1436 | 0,293 |
| 26 | Δspo0A | 16 | 4 | 3,362 | 1,0583 | 0,0035 |
| 27 | Δspo0A | 17 | 4 | 3,3627 | 0,9363 | 0,0015 |
| 28 | Δspo0A | 18 | 4 | 3,3622 | 0,7281 | 0,0014 |
| 29 | WT | 19 | 6 | 3,6083 | 0,3955 | 0,3023 |
| 30 | WT | 20 | 6 | 3,6352 | 0,7539 | 0,3181 |
| 31 | WT | 21 | 6 | 3,5816 | 0,3178 | 0,2361 |
| 32 | Δspo0A | 22 | 6 | 3,362 | 0,4368 | 0,0003 |
| 33 | Δspo0A | 23 | 6 | 3,3617 | 0,3166 | 0,0021 |
| 34 | Δspo0A | 24 | 6 | 3,3623 | 0,27 | 0,002 |
| 35 | WT | 25 | 8 | 3,608 | 0,2137 | 0,2969 |
| 36 | WT | 26 | 8 | 3,6248 | 0,211 | 0,2841 |
| 37 | WT | 27 | 8 | 3,5875 | 0,2231 | 0,2302 |
| 38 | Δspo0A | 28 | 8 | 3,3617 | 0,4008 | 0,0021 |
| 39 | Δspo0A | 29 | 8 | 3,3618 | 0,2573 | 0,0024 |
| 40 | Δspo0A | 30 | 8 | 3,362 | 0,2788 | 0,0043 |
| 41 | WT | 31 | 10 | 4,2287 | 0,5979 | 0,7584 |
| 42 | WT | 32 | 10 | 4,2194 | 0,3429 | 0,7392 |
| 43 | WT | 33 | 10 | 4,077 | 0,1573 | 0,6277 |
| 44 | Δspo0A | 34 | 10 | 3,3619 | 0,3444 | 0,0028 |
| 45 | Δspo0A | 35 | 10 | 3,3638 | 0,4219 | 0,0041 |
| 46 | Δspo0A | 36 | 10 | 3,3612 | 0,1471 | 0,0007 |
| 47 | WT | 37 | 12 | 5,6285 | 0,2182 | 2,3988 |
| 48 | WT | 38 | 12 | 5,4863 | 0,1313 | 1,9336 |
| 49 | WT | 39 | 12 | 5,3654 | 0,2714 | 1,3862 |
| 50 | Δspo0A | 40 | 12 | 3,3628 | 0,2695 | 0,0005 |
| 51 | Δspo0A | 41 | 12 | 3,3623 | 0,2326 | 0,0026 |
| 52 | Δspo0A | 42 | 12 | 3,3621 | 0,2048 | 0,0019 |
| 53 | WT | 43 | 13 | 6,4126 | 0,281 | 2,8102 |
| 54 | WT | 44 | 13 | 6,4915 | 0,2105 | 2,9762 |
| 55 | WT | 45 | 13 | 6,6413 | 0,2621 | 3,3102 |
| 56 | Δspo0A | 46 | 13 | 3,3622 | 0,2293 | 0,0044 |
| 57 | Δspo0A | 47 | 13 | 3,3636 | 0,3227 | 0,0054 |
| 58 | Δspo0A | 48 | 13 | 3,3619 | 0,17 | 0,0045 |
| 59 | WT | 49 | 24 | 12,3954 | 0,8298 | 19,0947 |
| 60 | WT | 50 | 24 | 13,4494 | 0,9262 | 22,3906 |
| 61 | WT | 51 | 24 | 12,9805 | 0,7472 | 20,1303 |
| 62 | Δspo0A | 52 | 24 | 3,365 | 0,4212 | 0,0073 |
| 63 | Δspo0A | 53 | 24 | 3,3633 | 0,3219 | 0,0039 |
| 64 | Δspo0A | 54 | 24 | 3,3652 | 0,3344 | 0,0019 |
| 65 | WT | 55 | 30 | 12,19 | 1,1061 | 19,3215 |
| 66 | WT | 56 | 30 | 13,5131 | 0,8085 | 26,6209 |
| 67 | WT | 57 | 30 | 13,2348 | 0,9257 | 24,7174 |
| 68 | Δspo0A | 58 | 30 | 3,3644 | 0,2724 | 0,0025 |
| 69 | Δspo0A | 59 | 30 | 3,3646 | 0,3806 | 0,0032 |
| 70 | Δspo0A | 60 | 30 | 3,365 | 0,3712 | 0,0048 |
| 71 | WT | 61 | 36 | 14,8893 | 1,0443 | 39,1718 |
| 72 | WT | 62 | 36 | 11,903 | 0,6772 | 22,7609 |
| 73 | WT | 63 | 36 | 14,478 | 1,0126 | 38,219 |
| 74 | Δspo0A | 64 | 36 | 3,3647 | 0,28 | 0,0058 |
| 75 | Δspo0A | 65 | 36 | 3,3647 | 0,2527 | 0,004 |
| 76 | Δspo0A | 66 | 36 | 3,3651 | 0,256 | 0,0047 |
| 77 | WT | 67 | 48 | 11,7374 | 0,8677 | 31,5778 |
| 78 | WT | 68 | 48 | 12,2047 | 1,3417 | 33,9294 |
| 79 | WT | 69 | 48 | 12,3684 | 1,4612 | 37,3532 |
| 80 | Δspo0A | 70 | 48 | 3,3656 | 0,269 | 0,0093 |
| 81 | Δspo0A | 71 | 48 | 3,3648 | 0,2881 | 0,0074 |
| 82 | Δspo0A | 72 | 48 | 3,3669 | 0,4439 | 0,0048 |

SSC=Solvents Standard Curve. Acetone, Ethanol and Butanol were used in different concentrations for the Standard Curve. Isopropanol was used as Internal Standard. n.a=not applicable. **Supplementary Table 3. Raw data for pH values during growth of WT and Δ*spo0A C. beijerinckii* NCIMB 8052.**

| **Strain** | **Time** | **pH** |
| --- | --- | --- |
|  | **hours** |  |
| WT | 0 | 5,83 |
| WT | 0 | 5,84 |
| WT | 0 | 5,84 |
| WT | 2 | 5,85 |
| WT | 2 | 5,86 |
| WT | 2 | 5,85 |
| WT | 4 | 5,80 |
| WT | 4 | 5,82 |
| WT | 4 | 5,79 |
| WT | 6 | 5,55 |
| WT | 6 | 5,57 |
| WT | 6 | 5,59 |
| WT | 8 | 5,18 |
| WT | 8 | 5,21 |
| WT | 8 | 5,23 |
| WT | 10 | 5,01 |
| WT | 10 | 5,01 |
| WT | 10 | 5,04 |
| WT | 12 | 5,11 |
| WT | 12 | 5,12 |
| WT | 12 | 5,08 |
| WT | 13 | 5,18 |
| WT | 13 | 5,19 |
| WT | 13 | 5,15 |
| WT | 24 | 5,85 |
| WT | 24 | 5,79 |
| WT | 24 | 5,77 |
| WT | 30 | 5,77 |
| WT | 30 | 5,80 |
| WT | 30 | 5,74 |
| WT | 36 | 5,77 |
| WT | 36 | 5,78 |
| WT | 36 | 5,78 |
| WT | 48 | 5,64 |
| WT | 48 | 5,64 |
| WT | 48 | 5,84 |
| Δspo0A | 0 | 5,80 |
| Δspo0A | 0 | 5,78 |
| Δspo0A | 0 | 5,77 |
| Δspo0A | 2 | 5,80 |
| Δspo0A | 2 | 5,74 |
| Δspo0A | 2 | 5,75 |
| Δspo0A | 4 | 5,64 |
| Δspo0A | 4 | 5,63 |
| Δspo0A | 4 | 5,65 |
| Δspo0A | 6 | 5,31 |
| Δspo0A | 6 | 5,35 |
| Δspo0A | 6 | 5,38 |
| Δspo0A | 8 | 4,95 |
| Δspo0A | 8 | 4,99 |
| Δspo0A | 8 | 5,01 |
| Δspo0A | 10 | 4,73 |
| Δspo0A | 10 | 4,81 |
| Δspo0A | 10 | 4,80 |
| Δspo0A | 12 | 4,66 |
| Δspo0A | 12 | 4,67 |
| Δspo0A | 12 | 4,67 |
| Δspo0A | 13 | 4,63 |
| Δspo0A | 13 | 4,59 |
| Δspo0A | 13 | 4,62 |
| Δspo0A | 24 | 4,53 |
| Δspo0A | 24 | 4,51 |
| Δspo0A | 24 | 4,51 |
| Δspo0A | 30 | 4,51 |
| Δspo0A | 30 | 4,52 |
| Δspo0A | 30 | 4,50 |
| Δspo0A | 36 | 4,55 |
| Δspo0A | 36 | 4,49 |
| Δspo0A | 36 | 4,51 |
| Δspo0A | 48 | 4,52 |
| Δspo0A | 48 | 4,52 |
| Δspo0A | 48 | 4,50 |

**Supplementary Table 4. Raw data for OD_600_ values during growth of WT and Δ*spo0A C. beijerinckii* NCIMB 8052.**

| **Strain** | **Time** | **ΟD_600_** |
| --- | --- | --- |
|  | **hours** |  |
| WT | 0 | 0,00 |
| WT | 0 | 0,01 |
| WT | 0 | 0,01 |
| WT | 2 | 0,00 |
| WT | 2 | 0,00 |
| WT | 2 | 0,01 |
| WT | 4 | 0,02 |
| WT | 4 | 0,02 |
| WT | 4 | 0,02 |
| WT | 6 | 0,06 |
| WT | 6 | 0,05 |
| WT | 6 | 0,04 |
| WT | 8 | 0,11 |
| WT | 8 | 0,11 |
| WT | 8 | 0,10 |
| WT | 10 | 0,17 |
| WT | 10 | 0,17 |
| WT | 10 | 0,17 |
| WT | 12 | 0,21 |
| WT | 12 | 0,22 |
| WT | 12 | 0,22 |
| WT | 13 | 0,26 |
| WT | 13 | 0,26 |
| WT | 13 | 0,27 |
| WT | 24 | 0,39 |
| WT | 24 | 0,40 |
| WT | 24 | 0,37 |
| WT | 30 | 0,44 |
| WT | 30 | 0,44 |
| WT | 30 | 0,42 |
| WT | 36 | 0,43 |
| WT | 36 | 0,44 |
| WT | 36 | 0,39 |
| WT | 48 | 0,37 |
| WT | 48 | 0,38 |
| WT | 48 | 0,40 |
| Δspo0A | 0 | 0,01 |
| Δspo0A | 0 | 0,01 |
| Δspo0A | 0 | 0,01 |
| Δspo0A | 2 | 0,01 |
| Δspo0A | 2 | 0,01 |
| Δspo0A | 2 | 0,01 |
| Δspo0A | 4 | 0,03 |
| Δspo0A | 4 | 0,02 |
| Δspo0A | 4 | 0,03 |
| Δspo0A | 6 | 0,07 |
| Δspo0A | 6 | 0,07 |
| Δspo0A | 6 | 0,07 |
| Δspo0A | 8 | 0,14 |
| Δspo0A | 8 | 0,13 |
| Δspo0A | 8 | 0,12 |
| Δspo0A | 10 | 0,21 |
| Δspo0A | 10 | 0,22 |
| Δspo0A | 10 | 0,20 |
| Δspo0A | 12 | 0,28 |
| Δspo0A | 12 | 0,24 |
| Δspo0A | 12 | 0,23 |
| Δspo0A | 13 | 0,26 |
| Δspo0A | 13 | 0,28 |
| Δspo0A | 13 | 0,25 |
| Δspo0A | 24 | 0,24 |
| Δspo0A | 24 | 0,23 |
| Δspo0A | 24 | 0,23 |
| Δspo0A | 30 | 0,24 |
| Δspo0A | 30 | 0,23 |
| Δspo0A | 30 | 0,24 |
| Δspo0A | 36 | 0,20 |
| Δspo0A | 36 | 0,19 |
| Δspo0A | 36 | 0,21 |
| Δspo0A | 48 | 0,25 |
| Δspo0A | 48 | 0,25 |
| Δspo0A | 48 | 0,24 |

**Supplementary Table 5. Oligonucleotides used in this study.**

| **Oligo ID** | **Oligo sequence (5’ to 3’)** | **Description** |
| --- | --- | --- |
| **Oligonucleotides for the amplification of homologous arms** | | |
| BG15796 | GAGATCTCCATGGACGCGTGACGTAATATAGTATTAATTTATGGTGTTATATTATATAAAAG | 500 bp homology arms upstream of *spo0A*, forward |
| BG14151 | ATTTTTTCTCTCCTTTTGTCC | 500 bp homology arms upstream of *spo0A*, reverse |
| BG14152 | GACAAAAGGAGAGAAAAAATTTGGCTTGAAGTAACATG | 500 bp homology arms downstream of *spo0A*, forward |
| BG14153 | CCGGGGATCCTCTAGAGTCGTTTGAAATATTATGACCTTTTTG | 500 bp homology arms downstream of *spo0A*, reverse |
| BG16051 | TCTCCATGGACGCGTGACGTACATATTCAACAGGTTCTTG | 500 bp homology arms upstream of *upp*, forward |
| BG16052 | TATTATTCCTCCAAGTTTGC | 500 bp homology arms upstream of *upp*, reverse |
| BG16053 | GCAAACTTGGAGGAATAATATTAGTAATTTATTAGAATTAAAAGCTATC | 500 bp homology arms downstream of *upp*, forward |
| BG16054 | CCGGGGATCCTCTAGAGTCGTCCTGCTTTGAATCCCTTAT | 500 bp homology arms downstream of *upp*, reverse |
| BG16736 | TCTCCATGGACGCGTGACGTCATGAAACCCTGCTGATAATG | 500 bp homology arms upstream of *Cbei_1291*, forward |
| BG16737 | GATTTCTATAAATATTTACTGCCTAATAATCAATAAAATTTATTCGGG | 500 bp homology arms upstream of *Cbei_1291*, reverse |
| BG16738 | AGTAAATATTTATAGAAATCGTAGGTAATGTG | 500 bp homology arms downstream of *Cbei_1291*, forward |
| BG16739 | CCGGGGATCCTCTAGAGTCGGTTAAATGAATTTTTATGCTCATATAAAAAAGTAATAG | 500 bp homology arms downstream of *Cbei_1291*, reverse |
| BG18279 | TCTCCATGGACGCGTGACGTGTGAAAATAAAATAGCTTCAAATTATGAG | 500 bp homology arms upstream of *Cbei_3238*, forward |
| BG18280 | CGCATACATCTCCCTTTTG | 500 bp homology arms upstream of *Cbei_3238*, reverse |
| BG18281 | ACAAAAGGGAGATGTATGCGTGTTAATGAGAAAAGATACAAC | 500 bp homology arms downstream of *Cbei_3238*, forward |
| BG18282 | CCGGGGATCCTCTAGAGTCGAATTATATCATACTTTCAGTAATACT | 500 bp homology arms downstream of *Cbei_3238*, reverse |
| BG16752 | TCTCCATGGACGCGTGACGTATATACTATTAACTAGAATAAATAATACTAAAGGAAG | 500 bp homology arms upstream of *Cbei_3932*, forward |
| BG16753 | TATAATAACCTCCAAAAAACTATCTATAATC | 500 bp homology arms upstream of *Cbei_3932*, reverse |
| BG16754 | GTTTTTTGGAGGTTATTATACCGCATGATTCATTAATTTGG | 500 bp homology arms downstream of *Cbei_3932*, forward |
| BG16755 | CCGGGGATCCTCTAGAGTCGGCATTAGGCCCGATTTTC | 500 bp homology arms downstream of *Cbei_3932*, reverse |
| **Oligonucleotides for knockout confirmation** | | |
| BG16483 | GGAATATAAAATAAACATAGGG | Δspo0A, forward |
| BG16484 | TTTCGCATACTATAATCCAC | Δspo0A, reverse |
| BG16862 | AATTGGATGCGATCATGGTG | Δupp, forward |
| BG16863 | ACTATTCTTAGAGAGTCATCGTCTTC | Δupp, reverse |
| BG18948 | GTTTTAACTTGTCTTTCCTCTC | ΔCbei_1291, forward |
| BG18949 | GATCCTTCTTTAATTCAGGG | ΔCbei_1291, reverse |
| BG18938 | CGTGTATCTGCAATCAGTTTTG | ΔCbei_3238, forward |
| BG18939 | CTCCCACCTATGTAATTTATTG | ΔCbei_3238, reverse |
| BG18936 | CAGTTAGGGATTTGAAACATACATG | ΔCbei_3932, forward |
| BG18937 | AAACCTAGCATCTAATGATTTAG | ΔCbei_3932, reverse |
| **Oligonucleotides for spacer insertion into pCOMA plasmid series; Spacers are underlined** | | |
| BG15843 | AGGTCTCATAGATCAGTATATTAAATGATTATCGTCTAAGAGACCA | spo0A spacer insertion through Golden Gate, forward |
| BG15844 | TGGTCTCTTAGACGATAATCATTTAATATACTGATCTATGAGACCT | spo0A spacer insertion through Golden Gate, reverse |
| BG16041 | AGGTCTCATAGATAACCTGTTTTTTCATCTCTTGTCTAAGAGACCA | upp1 spacer insertion through Golden Gate, forward |
| BG16042 | TGGTCTCTTAGACAAGAGATGAAAAAACAGGTTATCTATGAGACCT | upp1 spacer insertion through Golden Gate, reverse |
| BG16756 | AGGTCTCATAGATATCCGGCGCGCATCTTTCTCGTCTAAGAGACCA | Cbei_1291 spacer insertion through Golden Gate, forward |
| BG16757 | TGGTCTCTTAGACGAGAAAGATGCGCGCCGGATATCTATGAGACCT | Cbei_1291 spacer insertion through Golden Gate, reverse |
| BG18319 | AGGTCTCATAGATAAGAGGAACGTGCAATACGAGTCTAAGAGACCA | Cbei_3238 spacer insertion through Golden Gate, forward |
| BG18320 | TGGTCTCTTAGACTCGTATTGCACGTTCCTCTTATCTATGAGACCT | Cbei_3238 spacer insertion through Golden Gate, reverse |
| BG16762 | AGGTCTCATAGATTGACGCCCCATATATCTAACGTCTAAGAGACCA | Cbei_3932 spacer insertion through Golden Gate, forward |
| BG16763 | TGGTCTCTTAGACGTTAGATATATGGGGCGTCAATCTATGAGACCT | Cbei_3932 spacer insertion through Golden Gate, reverse |
| BG18724 | AGGTCTCATAGATCAGTATATTAAATGATTATCGTCTAAGAACTTTAAATAATTTCTACTGTTGTAGATAACCTGTTTTTTCATCTCTTGTCTAAGAGACCA | spo0A-upp1 spacer insertion through Golden Gate, forward |
| BG18725 | TGGTCTCTTAGACAAGAGATGAAAAAACAGGTTATCTACAACAGTAGAAATTATTTAAAGTTCTTAGACGATAATCATTTAATATACTGATCTATGAGACCT | spo0A-upp1 spacer insertion through Golden Gate, reverse |
| BG18726 | AGGTCTCATAGATAACCTGTTTTTTCATCTCTTGTCTAAGAACTTTAAATAATTTCTACTGTTGTAGATCAGTATATTAAATGATTATCGTCTAAGAGACCA | upp1-spo0A spacer insertion through Golden Gate, forward |
| BG18727 | TGGTCTCTTAGACGATAATCATTTAATATACTGATCTACAACAGTAGAAATTATTTAAAGTTCTTAGACAAGAGATGAAAAAACAGGTTATCTATGAGACCT | upp1-spo0A spacer insertion through Golden Gate, reverse |
| BG18728 | AGGTCTCATAGATCAGTATATTAAATGATTATCGTCTAAGAACTTTAAATAATTTCTACTGTTGTAGATTAAAATCCTTTGAACCTGTTGTCTAAGAGACCA | spo0A-upp2 spacer insertion through Golden Gate, forward |
| BG18729 | TGGTCTCTTAGACAACAGGTTCAAAGGATTTTAATCTACAACAGTAGAAATTATTTAAAGTTCTTAGACGATAATCATTTAATATACTGATCTATGAGACCT | spo0A-upp2 spacer insertion through Golden Gate, reverse |
| BG18730 | AGGTCTCATAGATTAAAATCCTTTGAACCTGTTGTCTAAGAACTTTAAATAATTTCTACTGTTGTAGATCAGTATATTAAATGATTATCGTCTAAGAGACCA | upp2-spo0A spacer insertion through Golden Gate, forward |
| BG18731 | TGGTCTCTTAGACGATAATCATTTAATATACTGATCTACAACAGTAGAAATTATTTAAAGTTCTTAGACAACAGGTTCAAAGGATTTTAATCTATGAGACCT | upp2-spo0A spacer insertion through Golden Gate, reverse |

**Supplementary Table 6. Knockout strains generated in this study.**

| Knockout name | Benchling link |
| --- | --- |
| *ΔCbei_0408-upp* | <https://benchling.com/s/seq-1JOwdJt8QWOzYhCDVSTl?m=slm-4JyiiWkuvvRC1rhpac4c> |
| Δ*Cbei_1291* | <https://benchling.com/s/seq-51mieTUESpOi1YjGlySN?m=slm-LklOYAluMlSUJB9Vs7P8> |
| Δ*Cbei_1712_spo0A* | <https://benchling.com/s/seq-urQiulVV7bnnWSpFoAwz?m=slm-eUXfGmPF5u2sF5NuTqiY> |
| Δ*Cbei_3238* | <https://benchling.com/s/seq-J8n9JPZdYYAFZWkztce4?m=slm-Qe2GaA2L75nmhpdaXVGn> |
| Δ*Cbei_3932* | <https://benchling.com/s/seq-N60RTFAV4KA28JkzX1Aj?m=slm-IDRoJQUoZQknBmZeZBhF> |
